# Dynamics of Global Gene Expression and Regulatory Elements in Growing Brachypodium Root System

**DOI:** 10.1038/s41598-020-63224-z

**Published:** 2020-04-27

**Authors:** Aaron J. Ogden, Thomas W. Wietsma, Tanya Winkler, Yuliya Farris, Gabriel L. Myers, Amir H. Ahkami

**Affiliations:** Environmental Molecular Sciences Laboratory (EMSL), Pacific Northwest National Laboratory (PNNL), Richland, Washington USA

**Keywords:** Plant development, Plant sciences, Plant molecular biology

## Abstract

Root systems are dynamic and adaptable organs that play critical roles in plant development. However, how roots grow and accumulate biomass during plant life cycle and in relation to shoot growth phenology remains understudied. A comprehensive time-dependent root morphological analysis integrated with molecular signatures is then required to advance our understanding of root growth and development. Here we studied *Brachypodium distachyon* rooting process by monitoring root morphology, biomass production, and C/N ratios during developmental stages. To provide insight into gene regulation that accompanies root growth, we generated comprehensive transcript profiles of Brachypodium whole-root system at four developmental stages. Our data analysis revealed that multiple biological processes including trehalose metabolism and various families of transcription factors (TFs) were differentially expressed in root system during plant development. In particular, the AUX/IAA, ERFs, WRKY, NAC, and MADS TF family members were upregulated as plant entered the booting/heading stage, while ARFs and GRFs were downregulated suggesting these TF families as important factors involved in specific phases of rooting, and possibly in regulation of transition to plant reproductive stages. We identified several Brachypodium candidate root biomass-promoting genes and *cis*-regulatory elements for further functional validations and root growth improvements in grasses.

## Introduction

Roots are the central core of the plant system which play a critical role during plant growth and development. Although root size and shape can be modified by environmental factors, the genotype and internal phenotype of an individual (at cellular and molecular levels) sets the limits within which such modification of growth and development can occur^1^. Our knowledge of the physiological basis underlying root growth and development and the relevant variations in belowground biomass production is poor, but should start with providing a basic understanding of the phenotypic and molecular determinants of global root architecture over the course of plant lifetime.

Grasses are critical components of providing global food and bioenergy. Improving the productivity of monocotyledonous flowering crops like wheat, rice, sorghum, etc. (Poaceae family) is therefore vital to keep pace with population growth. This requires a fundamental understanding of molecular physiology of organ development (e.g. leaf, flower, root, etc.) during the crop life cycle. Recently, *Brachypodium distachyon* (Poaceae, henceforth Brachypodium) has been chosen as a model system to study different aspects of temperate grasses because of its short lifecycle, relatively simple diploid genome^2^, and manageable growth requirements^3^. The vegetative and reproductive growth stages of *B. distachyon* have been thoroughly studied based on phenological characteristics of above-ground tissues^4^. Our limited knowledge in the area of grass root biology is partly due to the complex fibrous root system characteristic of monocots compared to the relatively simple taproot architecture in dicots like Arabidopsis. In this context, Brachypodium displays all the characteristics of a monocotyledon root system; however, its complexity is minimal compared to many other strategic food and bioenergy crops^5^. This makes Brachypodium an excellent and tractable model to study Root System Architecture (RSA) in grasses. The use of Brachypodium to accelerate the identification of root-related genes and markers and explore routes to translate these discoveries to other crops has been described^6^. Detailed characteristics of *B. distachyon* RSA during principal developmental stages has been reported, including root length and depth, as well as relationships between RSA and number of tillers and shoot biomass^4^. The timing of the emergence of Brachypodium roots relative to above-ground developmental stages has been described and is shown to be similar to that of wheat^7^. While the morphological attributes of Brachypodium root system have been thoroughly investigated, little is known about the molecular basis of root growth that govern these changes. Moreover, the molecular biology of root branching is not well understood in cereals including Brachypodium as a model grass.

Changes in RSA resulting in deeper, wider or thicker roots are controlled by complex interactions among tens to hundreds of genes^8^. To our knowledge, though, only one gene has been characterized to date that modulates a relatively subtle shift to root architecture in a monocot, DEEPER ROOTING 1 (*DRO1*) as a major quantitative trait loci (QTL) for deeper rooting in rice^9^. However, it is well-documented that almost all aspects of plant growth and development (including root formation) are governed in part by Transcription Factors (TFs)^8,10^. TFs activate or repress the expression of target genes both spatially and temporally, through the specific binding of *cis*-regulatory elements (CREs or motifs) present in their promoters. To regulate diverse and complex cellular processes relevant to different physiological pathways, plants have evolved a repertoire of over 50 TF families. Bioinformatics analyses of model plants such as *Arabidopsis thaliana*, *Oryza sativa*, and *Brachypodium distachyon* suggest each species encodes 1773^11^, 2,516^12^ TFs, and 1,687^12^ TFs, respectively. These regulatory factors including AUX/IAAs (Auxin/Indole Acetic Acid), ARFs (Auxin Responsive Factors), ERFs (Ethylene Response Factors), and NAC (NAM, ATAF1/2 and CUC2) have been suggested as major key players to better understand root development in response to external and internal growth regulators^10,13–22^. Additionally, recent efforts in plant motif discovery and curating reported CREs^11,23,24^ have been valuable, providing powerful hints into the role of TFs in regulating specific promoter sequences of interest. Few studies have conducted transcriptome and CRE analyses in revealing biologically relevant target genes and providing clues into the regulatory relationships of TFs in key abiotic stress responses in Brachypodium^25–27^. However, to date no study has characterized Brachypodium gene profiles and promoter architecture for identification of putative root regulatory modules and at a growth-stage dependent manner.

Most of the previous studies in the area of root biology focused on investigating the molecular basis of root development and cell differentiation in specific root zones, cell-types, and at single and very early seedling developmental stages^28^. Also, the basis of these works was primarily to study the response of root tissue to environmental signals (stressed condition) rather than to internal growth regulators (non-stressed condition). The main objective of this study was to determine the time-dependent gene expression profiles and dynamics of regulatory elements in *B. distachyon* whole-root system in relation to well-characterized aboveground developmental phases as leaf development, early tillering, late tillering and booting/heading stages. We demonstrated the utility of our dataset by identifying differential expressed genes and highlighting the most important TFs at each time point of root growth including the ones with possible roles in root branching. Our results provide the most comprehensive dataset of Brachypodium rooting process at the transcript level to date, and the identified putative root growth-promoting genes and regulatory elements in this work are potential targets to generate genetically modified crops for biomass increase in future studies.

## Results

### Morphology of Brachypodium RSA during growth stages

**Figure 1:**
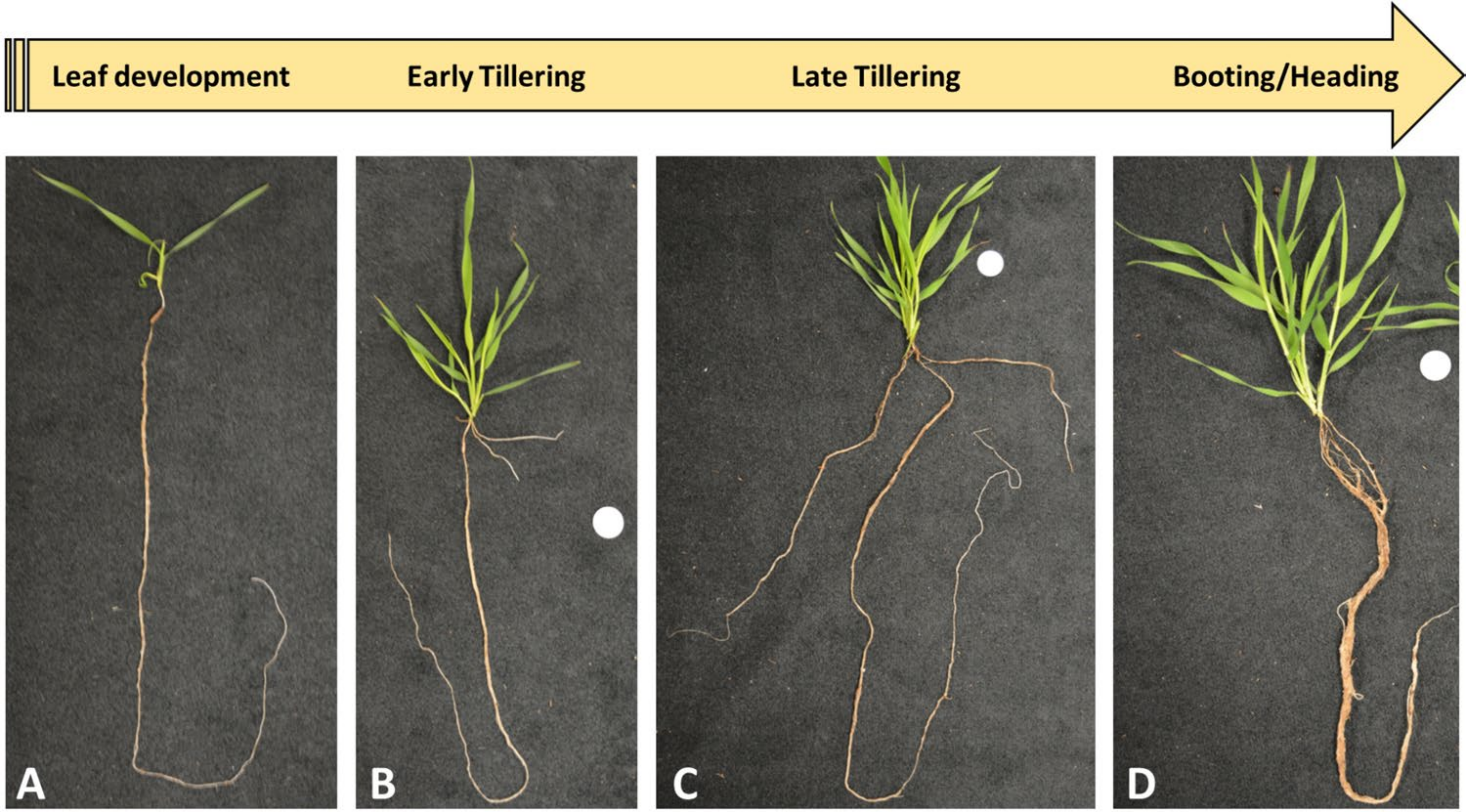
Plants representative of the different developmental stages used in this study. Plants were harvested at four developmental stages consisting of (**A**) leaf development stage, T1 (18 DPS); (**B**) early-tillering stage, T2 (25 DPS); (**C**) late-tillering stage, T3 (32 DPS); and (**D**) booting/heading stage, T4 (36 DPS). Scale disk diameter = 0.5”.

**Figure 2:**
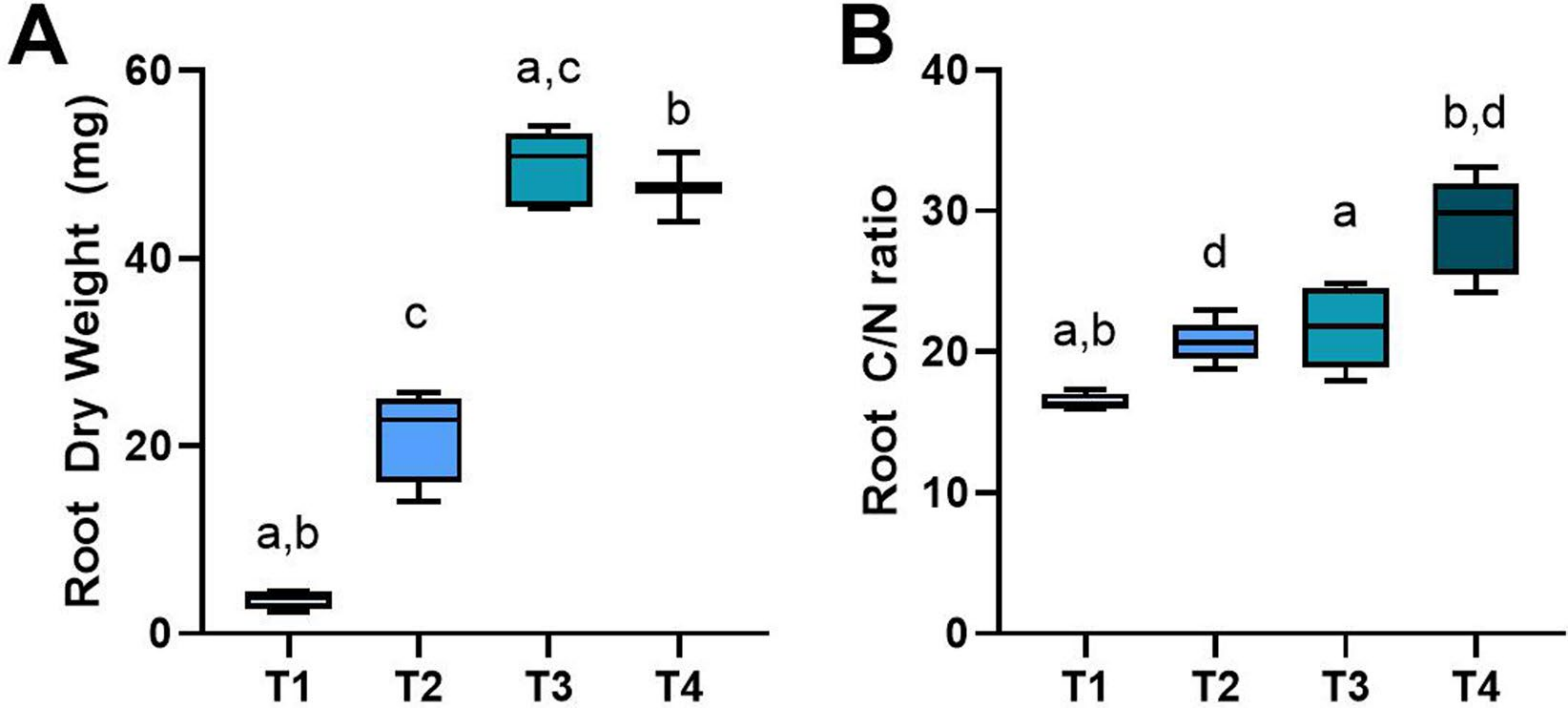
Root dry weight and carbon/nitrogen ratios. Brachypodium root dry weights (**A**) and carbon to nitrogen ratio (**B**) were collected at each timepoint throughout the experiment. Error bars represent minimum and maximum values. Both dry weight and carbon/nitrogen measurements resulted in a p-value < 0.005 via Kruskal-Wallis test. Further pairwise post-hoc tests were performed by Dunn’s test, with significant changes between T1-T3 (a), T1-T4 (b), T2-T3 (c), and T2-T4 (d) (p-value < 0.05).Each value is represented by the mean of three to five independent replicates (n = 4, 4, 5, and 3 for T1-T4, respectively).

We monitored Brachypodium root phenotypic changes during its primary four principal growth stages. Seedlings with two to three leaves at leaf development stage (T1) showed single primary root growth (Fig. 1A), while early tillering stage (T2) was the representative of developing primary nodal roots (coleoptile nodal roots in the hypocotyl region) (Fig. 1B). As plants produced more tillers at late tillering stage (T3), coleoptile nodal roots elongated, and branch roots started to form (Fig. 1C). When plants showed the very initial signs of transition into reproductive stage at booting stage (T4, emerging the head at the top of the growing shoot and swelling of the flag leaf sheath^4^), leaf nodal roots formed in the stem base area and elongated, and several branch roots developed making whole roots thicker below-ground (Fig. 1D). Root dry weight was significantly increased from 4 mg at T1 up to about 50 mg at T3 and T4 (Fig. 2A). Overall, we did not observe significant changes in mean root dry weight between T3 and T4 replicates (Although minor differences in dry weight and root patterning were observed between individual biological replicates within samples harvested in each time point.) To evaluate whether the biomass accumulation was reflected by a change of carbon to nitrogen ratios during rooting, and to evaluate the possible relationship between C/N ratios with temporal root gene expression profiles, total concentrations of carbon and nitrogen were measured (Supplemental Table S1). There was a slight increase in the carbon to nitrogen ratio at T2 and T3; however, a significant increase up to 2-fold at T4 was monitored compared to T1 (Fig. 2B).

### Comprehensive transcript profiles of the developing root

To identify important time-dependent regulators of root growth in response to internal growth stimuli, we generated comprehensive transcript profiles of Brachypodium whole-root system at different developmental stages (T1-T4, see above and Fig. 1). Summarized in supplemental Table S2, the Illumina-based paired end sequencing resulted in tens of millions of high-quality reads per sample that allowed for efficient alignment via Hisat2. The outcome of this procedure was the identification and abundance estimation of 21,764 genes that were subsequently normalized via DESeq. 2^29^ (Supplemental Fig. S3). Included in Supplemental Table S4, our highly curated dataset provides a novel and comprehensive resource of Brachypodium genes with possible roles in root development.

### qRT-PCR supports RNA Seq. findings

To validate the accuracy of our transcriptome gene-counts, we selected multiple genes for relative quantification using qRT-PCR. The partial least squares discriminant analysis (PLS-DA), hierarchical clustering analysis, and manual examination of the data (see below) indicated that most genes in T2 and T3 were at intermediate values between T1 and T4. We therefore chose to perform qPCR on T1 and T4 samples. As shown in supplemental Fig. S5, for 7 transcription factors belonging to ERF, NAC, ABA, MADS, and cytokinin families, the changes in RT-qPCR transcript abundances from T1 to T4 correlated well with corresponding normalized gene count estimations generated via RNA-seq as measured by Pearson correlation coefficient (PCC)^30,31^. One notable exception, Bradi_1g14230, showed a weak negative correlation (PCC = −0.22).

### PLS-DA and GO term enrichment analysis suggests multiple biological pathways involved in Brachypodium root growth and development

**Figure 3:**
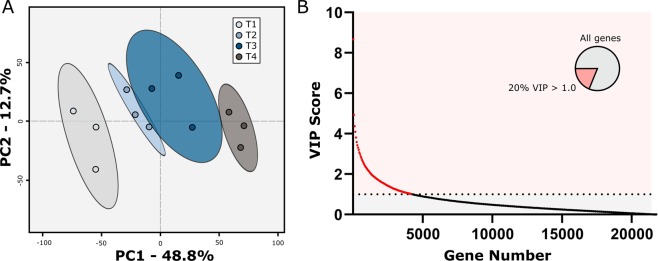
PLS-DA score plot and VIP plot. (**A**) Partial least squares discriminant analysis (PLS-DA) score plot generated from each timepoint. (**B**) Variable of importance in projection (VIP) scores of each gene used in the PLS-DA. Genes contributing meaningfully to the PLS-DA model with a PC1 VIP score >1.0 constitute 20% of the genes tested (inset pie-chart) and can be found in supplemental table S4.

To identify genes with potential roles in *B. distachyon* root growth and development, we employed both multivariate and univariate analyses. First, we performed PLS-DA using all 21,764 identified genes. Principal component 1 (PC1) of the score-plot resulting from this PLS-DA comprised 46.3% of data variation and was caused by differences between T1 and T4 (Fig. 3A). Samples T2 and T3 were located between T1 and T4 on the score-plot, suggesting the transcript abundance for most genes at T2 and T3 were intermediate between those observed at either T1 or T4. We then identified the genes that contribute to the large variation observed in PC1 by extracting genes with a high VIP score (Variable of Importance in the Projection score >1.0)^32–34^ (Fig. 3B). The resulting 4,247 genes were selected for gene ontology (GO) enrichment analysis^32,35^.

**Figure 4:**
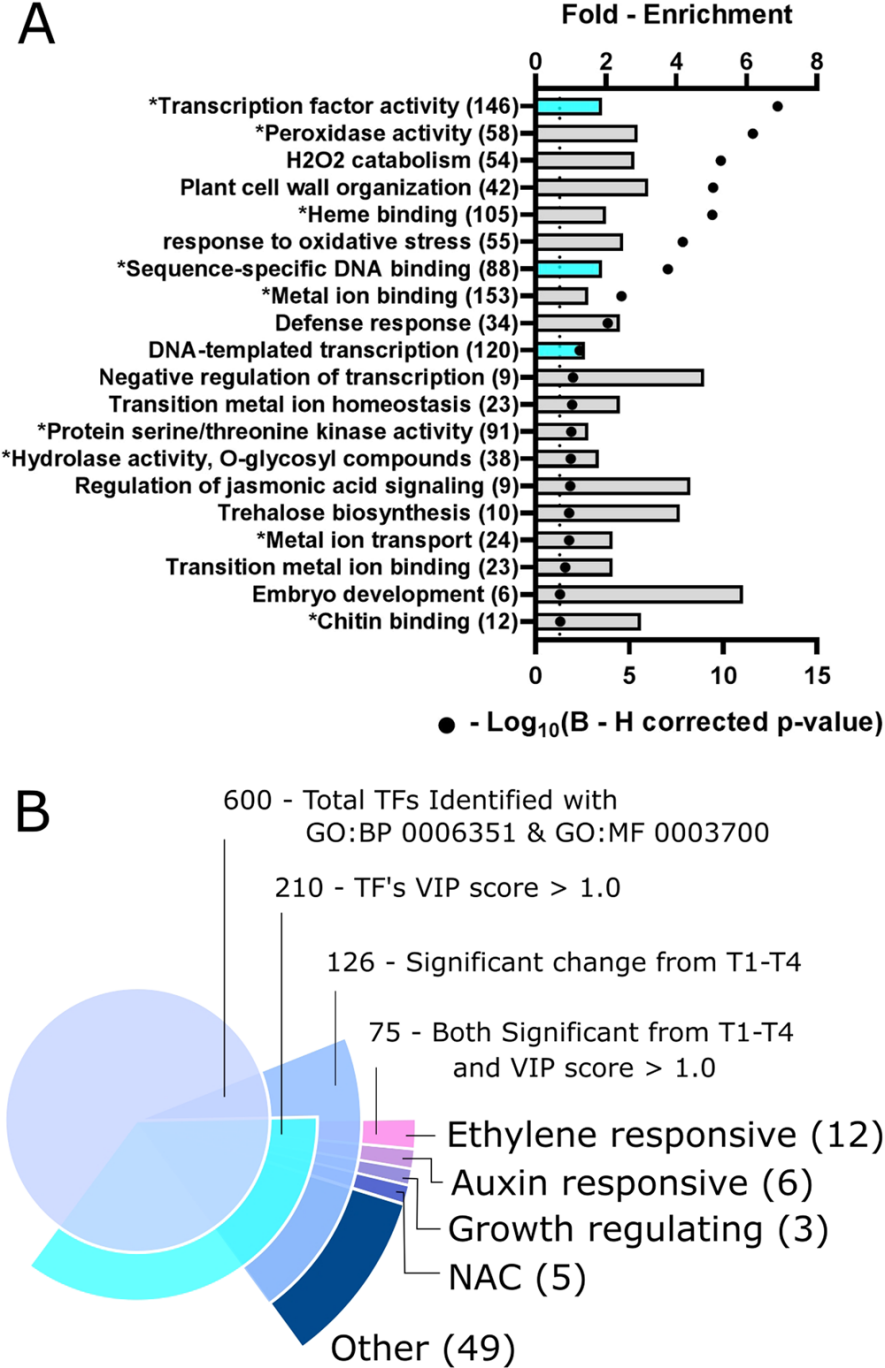
GO term enrichment and transcription factor summaries. (**A**) Bar graph representation of the Gene Ontology (GO) terms that are significantly over-represented among genes with a PLS-DA PC1 VIP score >1.0. The fold-enrichment and Benjamini-Hochberg (**B–H**) corrected p-values are shown as bars on the top x-axis and black circles on the bottom x-axis, respectively. Turquoise highlighted bars correspond to GO term 0006351 (top), 0043565 (middle), and 0003700 (bottom). The asterisk denotes a GO molecular function (MF) term, while all other terms are GO biological processes (BP). For reference, the vertical dotted line represents a B-H p-value of 0.05. (**B**) Expanding pie-chart summarizing the 600 non-redundant transcription factors identified in this study (GO:0006351 & 0003700). For both A and B, numbers within parentheses represent the number of genes contributing to that list. The 75 TFs can be found in supplemental Table S6.

To better understand the biological processes involved in root development, we performed an enrichment analysis using the gene ontology terms of genes with a high PLS-DA PC1 VIP score. As shown in Fig. 4A, multiple GO biological process and molecular function terms were significantly enriched among these genes using the DAVID bioinformatics resource (v6.8) (Benjamini-Hochberg corrected p-value < 0.05)^36^. Of major importance were the biological processes plant cell wall organization (GO:0009664) and DNA-templated transcription (GO:0006351) which were 3.2- and 1.4-fold enriched, respectively, within our dataset as compared to the Brachypodium genome background. Similarly, the GO molecular function terms transcription factor activity (GO: 0003700) and sequence-specific DNA binding (GO: 0043565) were both significantly 1.9 enriched (Fig. 4A, asterisks). For convenience, an annotated list of all genes belonging to each enriched GO term shown in Fig. 4A can be found in Supplemental Table S6, including their predicted function, gene count fold-change from T1 to T4, and t-test results. To highlight the most important trends in root gene expression observed during growth, we grouped the responsive genes into functional categories based on the GO terms. Here, we independently describe the transcriptional changes observed for each of these functional categories during root growth.

### Transcription factors are essential for the regulation of gene expression in growing Brachypodium root system

Our enrichment analysis strongly suggested that *B. distachyon* root development is governed in part by dynamic regulation of TFs. Our dataset of 21,764 genes contained abundance estimates for 600 likely TFs identified as having a GO biological process term of “DNA-templated transcription” (GO:0006351), or the GO molecular function terms “Transcription factor activity” (GO:0003700) and “sequence-specific DNA binding” (GO: 0043565). Over 200 of these TFs were found to have a high PC1 VIP score and therefore could be involved in Brachypodium root growth during plant development (Supplemental Table S6). This subset of TFs is comprised of multiple families including MADS-box, NAC, ARF, ERF, and WRKY, and others. Independent of the multivariate PLS-DA analysis, we observed 126 TFs whose gene count abundance changed significantly from T1 to T4 using the univariate t-test with a B-H adjusted p-value threshold (Fig. 4B). Not surprisingly, there is a large overlap of 75 TFs that were both differentially expressed between T1 and T4 (126 TFs) and had a PLS-DA PC1 VIP score (210 TFs). Although TFs identified by PLS-DA PC1 VIP scores are likely involved in Brachypodium rooting, these 75 TFs are the most likely contributors to root growth during the investigated time frame of plant development. (See Supplemental Table S7 for individual lists of each statistically identified TFs).

### Hierarchical clustering analysis classifies TFs into four distinct groups

**Figure 5:**
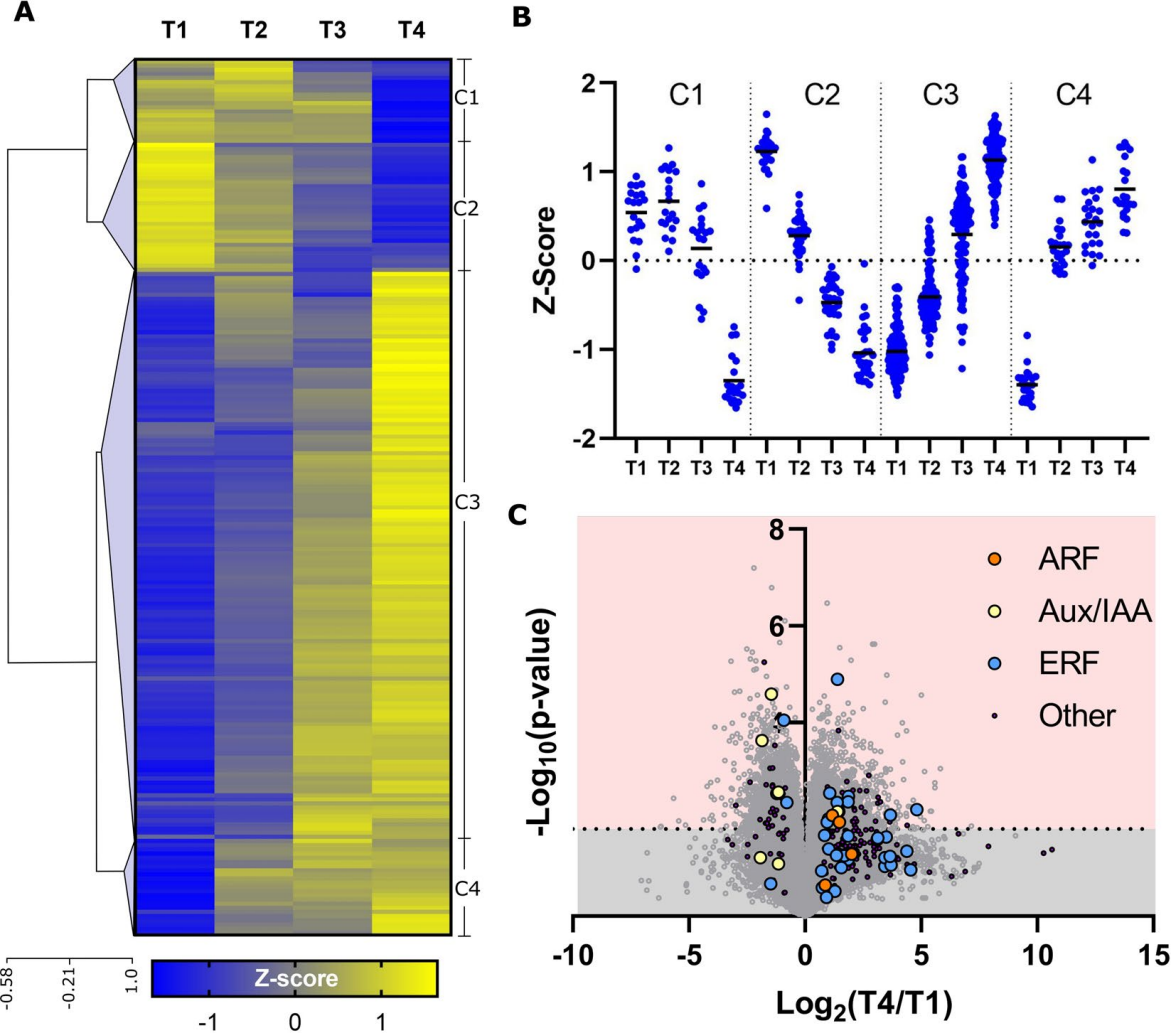
Transcription factor hierarchical clustering, cluster trends, and volcano plots. (**A**) Heatmap representation of a hierarchical clustering analysis using Z-scores for each of the 210 TFs with a high VIP score in PC1, resulting in 4 major clusters (C1-C4). (**B**) The apparent transcriptional regulation of TFs can be seen for each cluster. Solid black lines indicate the average Z-score value for all genes within each time point and cluster. (**C**) Volcano plot shows many of the 210 transcription factors (purple dots) are significantly differentially regulated from T1 to T4. Dotted line indicates the B-H adjusted p-value threshold.

While the PLS-DA identifies TFs whose transcript abundance change from T1 to T4 and are therefore likely involved in Brachypodium root growth, it does not describe whether the expression of those TFs increased or decreased over time. To better understand the differential regulation of TFs during root development, we performed a hierarchical clustering analysis (HCA) using 210 TFs with a high PC1 VIP score and generated a corresponding heatmap (Fig. 5A and Supplemental Table S7). The four clusters produced by our HCA suggested positive or negative roles for TFs during developmental stages (Fig. 5B). For example, cluster 1 (C1) contained TFs likely involved in early development from T1 to T3. Most TFs, however, belonged to cluster 2 (C2) and cluster 3 (C3), whose abundance changes in a linear fashion from T1 to T4, suggesting that most TFs were not highly specific or differentially regulated in any individual developmental stage. Finally, cluster 4 (C4) likely represented TFs that were involved in later development from T2 to T4.

### Phytohormone-related TFs are differentially regulated during rooting

Our PLS-DA identified 6 AUX/IAA or AUX/IAA-like genes, as well as 4 ARF or ARF-like genes that have a high VIP score >1 in PC1 (based on the VIP scores presented under column J in the Supplementary Table S7). Five of the six Aux/IAA TF family were identified in HCA cluster C2, suggesting a down-regulation of auxin-response IAA expression, including Bd1g14230, Bd1g09090, and Bd4g02580 which were significantly downregulated (B-H corrected p-value < 0.014). Conversely, all four members of the ARF TF family appear upregulated based on VIP score and were identified in cluster C3, including Bd4g17410 and Bd2g59480, both of which were significantly upregulated (B-H corrected p-value < 0.014).

We identified 35 TFs associated with ethylene signaling using PLS-DA (PC1 VIP score >1, supplemental Table S6), 33 of which belonged to the ERF or ERF-like TF families. Almost all (except 3 out of 33 ERFs) were identified in the HCA clusters C3 and C4, suggesting an increase in ERF expression during Brachypodium root growth. Almost 40% of the 33 ERFs identified in clusters C3 and C4 were significantly upregulated during root growth, (Supplemental Table S7). Three notable exceptions to this trend were the RAP2 subgroup of ERFs (accessions Bd5g24110v3, Bd1g45470v3, and Bd4g29010v3), each of which were significantly downregulated in T4 compared to T1 and were found in clusters C1 and C2 (Fig. 5C). The downregulation of these characteristically hypoxia-induced RAP2 TFs^37^ is consistent with the lack of hypoxic plant growth conditions during our experiment.

### WRKY, NAC and MADS TFs are upregulated, while GRF TFs are downregulated during root growth and development

A total of 31 WRKY-like TFs were identified that had a high VIP score greater than 1 in PC1 (based on the VIP scores presented under column J in the Supplementary Table S7). All but one belonged to clusters C3 and C4 and showed a 2- to 120- fold increase, indicating an upregulation of the WRKY TF family during *B. distachyon* root development. Despite their importance in the VIP model, only Bd2g18530, Bd2g53520, Bd4g28280, and Bd1g09170 were significantly upregulated (B-H corrected p-value < 0.014). Like WRKY TFs, the high expression levels (up to 9-fold increase) of all but 2 of the 19 detected NAC TFs of clusters C3 and C4 (with a high PC1 VIP score), indicated a NAC family-wide upregulation as the root grows from T1 to T4. Five of the 17 NAC TFs, including Bd2g57100, Bd1g76207, Bd2g03467, Bd4g44000, and Bd4g34157 were significantly upregulated in T4 (B-H corrected p-value < 0.014). In contrast with the WRKY, and NAC TFs, we identified 3 of the 10 known *B. distachyon* GRF TFs (Bd1g50597, Bd1g09900, and Bd4g16450), all of which were significantly 1.8- to 2.6-fold down-regulated in T4 compared to T1. Overall, we showed that the abundance of most TFs within a TF family changed in a similar family-wide manner over time.

### Promoter analysis identifies over-represented DNA elements in each cluster of identified TFs

We also analyzed 1000 bp from the promoter regions of each of the genes in each TF cluster (C1-C4, Fig. 5A) using Multiple Expectation Maximization for Motif Elicitation (MEME) program to identify strongly enriched growth stage-specific *cis*-regulatory DNA elements during rooting^38^. We found over-representation of total ten certain short DNA sequences in member gene promoter sequences in all clusters (Table 1). Four clusters had at least one significantly over-represented DNA element (E-value < 0.05). This may provide insight into the transcriptional circuitry^26^ that mediates the regulation of the gene clusters during plant development and rooting process. Using GOMo (Gene Onthology for Motifs) tool, we screened all promoters in each cluster to determine if any motif is significantly associated with genes linked to one or more GO terms^39^. Our results showed between one (the lowest) and 33 (the highest) GO term predictions for a given motif (Table 1 and Supplementary Fig. S8). To further study the possible biological roles of the motifs, we searched the degenerate consensus sequences of the significantly enriched elements identified by MEME against PLACE, a Database of Plant Cis-acting Regulatory DNA Elements. Consequently, seven out of the ten tested motifs revealed to have a similar signal sequence present in the PLACE database, suggesting possible roles of these already known motifs in Brachypodium rooting (Table 1). Enrichment of previously uncharacterized motifs including TCYCTCCCTYCC (cluster 3), WGCTAGCTAGCT (cluster 3), and TTCTKCYYCTCY (cluster 4), suggests that novel promoter elements may function in regulating Brachypodium root growth and development in a time-dependent manner.

**Table 1:** Promoter motif analysis of TFs belonging to different HCA clusters.

Cluster (n)	MEME Analysis		GOMo Analysis						PLACE Analysis		
	Logo	Sites	E-value	GP	GO:	GO #	GO Name	q-value	Factor name	Sig. Seq.	Site #
**Cluster 1**											
1 (18)		18	2E-05	11	BP	0045449	Regulation of Transcription	2E-03	NODCON2GM	CTCTT	S000462
					BP	0048364	Root Development	8E-03	OSE2ROOTNODULE	CTCTT	S000468
					BP	0016123	Xanthophyll Biosynthetic Process	1E-03			
		18	6E-05	1	MF	0003700	Transcription Factor Activity	1E-03	ANAERO1CONSENSUS	(-) AAACAAA	S000477
**Cluster 2**											
2 (29)		20	3E-02	1	MF	0003700	Transcription Factor Activity	1E-03	ANAERO1CONSENSUS	AGCAGC	S000478
**Cluster 3**											
3 (124)		92	6E-28	12	BP	0006355	Regulation of Transcription, DNA	2E-02	MARTBOX	TTWWTTWTT	S000067
					BP	0007623	Circadian Rhythm	6E-04			
					BP	0009753	Response to Jasmonic Acid	2E-03			
		124	1E-18	33	BP	0006355	Regulation of Transcription, DNA	8E-05	DOFCOREZM	AAAG	S000265
					BP	0007169	TMR Tyrosine Kinase signaling	8E-05			
					BP	0006468	Protein Amino Acid Phosphorylation	8E-05			
		76	4E-10	27	BP	0006355	Regulation of Transcription, DNA	1E-04	NA		
					BP	0010158	Abaxial Cell Fate Specification	6E-03			
					BP	0009744	Response to Sucros Stimulus	1E-02			
		25	2E-07	2	MF	0003700	Transcription Factor Activity	1E-03	NA		
					CC	0012505	Endomembrane System	3E-02			
		44	3E-02	9	BP	0006355	Regulation of Transcription, DNA	3E-02	SORLREP3AT	(-) TGTATATAT	S000488
					BP	0005975	Carbohydrate Metabolic Process	1E-02			
					BP	0009733	Response to Auxin Stimulus	2E-04			
**Cluster 4**											
4 (21)		21	3E-04	33	BP	0006355	Regulation of Transcription, DNA	7E-05	CTRMCAMV35S	TCTCTCTCT	S000460
					BP	0007623	Circadian Rhythm	7E-05			
					BP	0009744	Response to Sucrose Stimulus	3E-03			
		21	4E-02	9	BP	0007169	TMR Kinase Signaling	2E-02	NA		
					MF	0003700	Transcription Factor Activity	8E-03			
					MF	0004674	Protein Serine/Threonine Kinase	1E-02			

TMR, Tyrosine Membrane Receptor; GP, # of GO terms Predicted by GOMo; n, number of contributing promoters; Sig. Seq., Signal Sequence; MF, Molecular Function; BP, Biological Process; CC, Cellular Component.

### Expression of expansins and peroxidases decrease during later stages of rooting

In addition to TFs, gene ontology enrichment analysis of genes with a high VIP score revealed a possible role for genes involved in the biological process “plant cell wall organization” (GO: 0009664) during Brachypodium root growth and development (Fig. 4A). Among the 42 genes within this category, we identified 12 putative expansins, 9 of which significantly decreased in abundance during root growth from T1 to T4 (supplemental Table S6). The expansin protein family is associated with the loosening of plant cell walls to facilitate cell expansion in the root elongation zone, as well as the initiation of root hairs^40–42^. Our data suggests that cell wall loosening is possibly less frequent as the root develops in Brachypodium. This interpretation stems from observations that expansins are highly expressed in the elongation zone near the root tip^43^, a region that is less represented in our whole-root study at later timepoints as root size increased (Fig. 1). In addition to expansins, the remaining 30 genes associated with the GO term “plant cell wall organization” were exclusively peroxidases. With few exceptions, these peroxidases exhibited similar trend of down-regulation during later root development, with 15 of 30 being significantly downregulated in T4 compared to T1, suggesting a decrease in hydrogen peroxide generation and potentially a lower demand for lignin formation and cell wall rigidification^44,45^.

### Jasmonate signaling is repressed during root growth and development

Gene ontology enrichment showed a significant 4.4-fold enrichment of genes with the biological process “regulation of jasmonic acid mediated signaling” (GO: 2000022). All 9 genes in this enriched group were found as members of the TIFY or TIFY-like gene family, including Bd1g21490v3, Bd4g31240v3, and Bd1g72590v3, each of which were significantly upregulated in T4 compared to T1. The TIFY family are repressors of the JA signaling pathway involved in root growth^46,47^, and their overexpression has been linked to increased shoot and root growth, as well as a decreased time to the heading stage (just before flowering) in rice^48^. Our data suggests that JA-mediated growth repression is decreased by upregulation of TIFY domain containing genes during root growth as Brachypodium plants develop and enter reproductive growth stages.

### Trehalose metabolism changes at transcript levels in roots during plant development

Our enrichment analysis of genes with a high PC1 VIP score identified a significant 4-fold enrichment of genes associated with trehalose metabolism. These 10 genes consisted of 6 trehalose phosphate synthases (TPSs) and 4 trehalose phosphate phosphatases (TPPs). Both genes function in the trehalose biosynthesis pathway by two sequential steps using trehalose-6-phosphate as an intermediate^49,50^. Our data showed a significant increase in expression of the TPSs Bd4g41580 and Bd1g69420 in T4 compared to T1, suggesting altered root trehalose metabolism during Brachypodium developmental stages, possibly as a signaling pathway triggering root growth responses^51^.

## Discussion

Despite the critical roles of root system during plant life cycle, our understanding of the temporal increase in size and mass of whole RSA at the molecular level is fragmentary. Understanding the underlying biological principles guiding root growth and proliferation requires knowledge of transcriptional changes during plant developmental stages^52^. The study of mutant phenotypes has proven to be a useful tool in the study of root systems, although the clear majority of such experiments have focused on a single growth stage and rooting zones (e.g. region of cell division or elongation zone), or the mutant root’s capacity to respond to abiotic stresses (like water deficiency and soil salinity), and to exogenous hormone or sugar treatments. Although these studies have provided noticeable and valuable information about root structure and physiology, little is known about the genetic regulation of global root architecture under non-stressed conditions, and solely in response to time-dependent internal growth stimuli. In this study, we generated comprehensive transcript profiles during early four developmental stages of the model grass Brachypodium. Each time point was chosen to capture specific root phenotypes of RSA, as well as the transition from predominantly vegetative growth to reproductive growth. To identify genes that are likely involved in root growth we employed multivariate analyses like PLS-DA and HCA, hypergeometric gene ontology enrichment tests, as well as univariate t-tests focused on differences between T1 and T4. Consequently, a large number of TFs were significantly enriched among the identified genes with a high VIP score in PC1, many of which were significantly differentially regulated during root growth.

**Figure 6:**
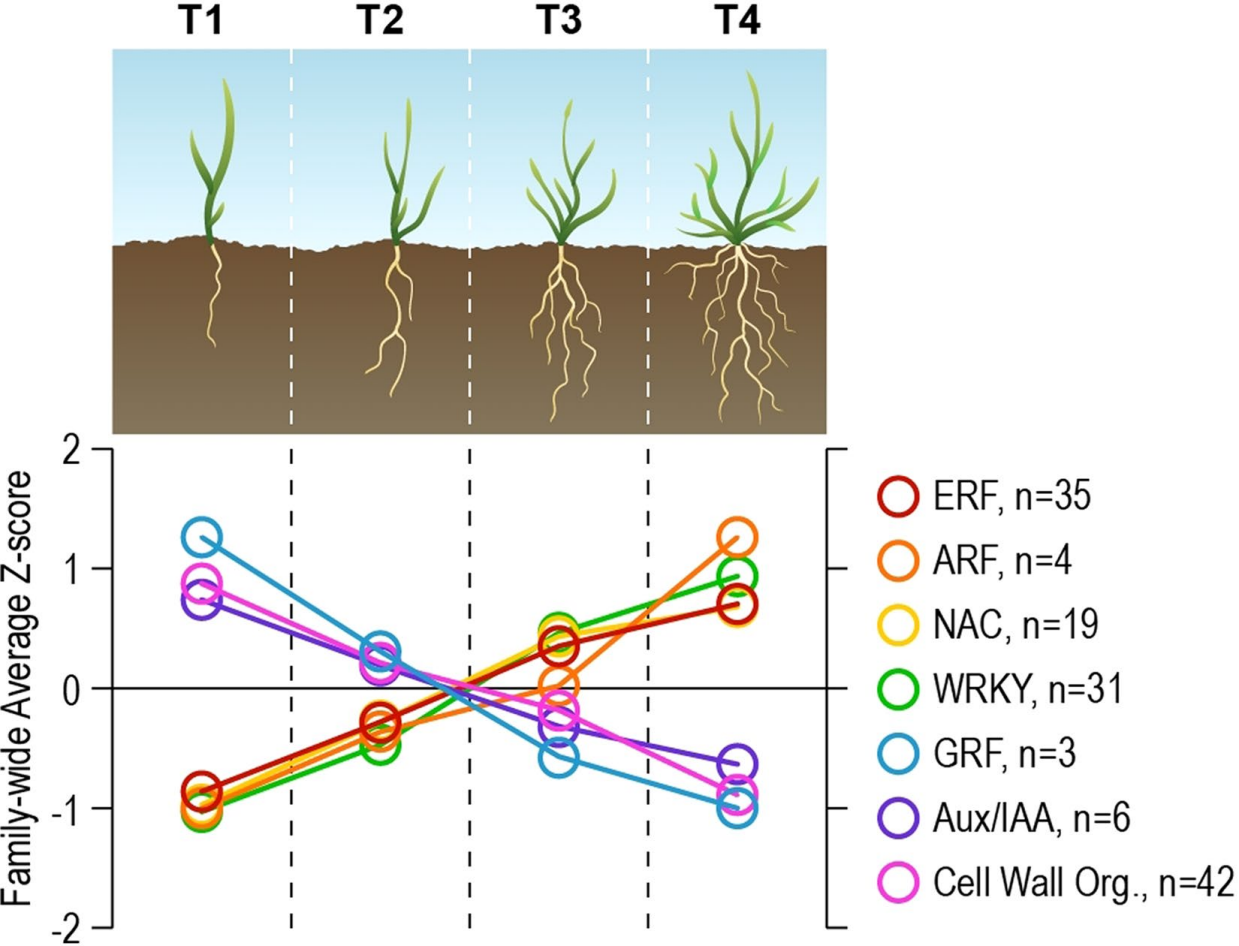
Family-wide trends in gene expression during Brachypodium growth. Each circle represents the average Z-score calculated using Z-scores of all TFs within a given family at that timepoint (or biological process, in the case of Cell Wall Organization). The number of genes contributing to each average Z-score is shown on the right. Org, Organization. (T1): leaf development stage, (T2): early-tillering stage, (T3): late-tillering stage, and (T4): booting/heading stage.

With the advent of high-throughput omics technologies, several studies of TFs identification and characterization have been conducted in Arabidopsis^53,54^ and rice^55^. However, much less information is available for Brachypodium. Besides, our knowledge of the TF dynamics controlling the entire root proliferation and growth during crop life cycle remains poor and fragmentary. Analysis of our dataset revealed wide trends in differential regulation within TF families, including AUX/IAAs, ARFs, and ERFs, suggesting an important role of auxin and ethylene regulatory networks in driving root growth in Brachypodium. Specifically, at later growth stages (T4) we observed a predominant down-regulation of AUX/IAA TFs concurrent with an up-regulation of both ARF and ERF TFs as compared to T1 (Fig. 6). The AUX/IAA TFs have been shown to function as transcriptional repressors of auxin responsive genes, while the opposite is true for ARF TFs^56^. The role of auxin in primary root length, lateral root formation and elongation has been documented^57–61^. This supports our finding on activation of auxin gene expression machinery in growing Brachypodium RSA, reflected by the phenotyping observations in T1-T4 (Figs. 1 and 2). Additional gene functional and biochemical studies are required to further understand the phase-dependent auxin (IAA) accumulation (spatial localization) and regulation and its interplay with other key root growth factors including expansins. It is documented that the primary transcriptomic effect associated with elevated auxin levels in root cells is changes in regulation of expansins^62^, which is coincident with our data (see above).

Plants control lateral root formation through multiple auxin-signaling modules^63^, which are known as pairs of strongly interacting Aux/IAA and ARF that regulate a subset of auxin response genes^64^. The *A. thaliana* genome encodes 29 AUX/IAAs and 23 ARFs^65–67^, and the *B. distachyon* genome encodes 25 AUX/IAAs and 24 ARFs^2^. This results in an extensive number of theoretically possible AUX/IAA and ARF combinations. Experimental analyses suggest preferential interactions between a subset of these Aux/IAA and ARF proteins in the process of lateral root development in Arabidopsis (e.g. IAA28-ARF5,6,7,8,19 or IAA14-ARF7,19 modules)^68^. However, the root branching and patterning of lateral roots are unique in monocots and our current knowledge on this issue is very limited and fragmentary^69,70^. In this work, we identified few ARF and IAA genes, suggesting several possible auxin-signaling modules (pairs) consisting of ARF4, ARF23 and IAA12, IAA13, IAA24, IAA31 (see the Supplemental Table S6, the 75 TFs sheet) with potential roles in root branching in T4 in Brachypodium.

The increase in auxin response typically causes an increase in the gaseous phytohormone ethylene response, as illustrated by many works describing the two closely connected pathways^71–74^. In this work, we observed a transcriptional increase in almost all detected ethylene response factors (ERFs) during root growth. Increased synthesis or signaling of ethylene has been shown to have an inhibitory effect on root branching in Arabidopsis because ethylene-insensitive mutants form more lateral roots^75,76^. Nevertheless, there is only limited knowledge about the role of this phytohormone during the formation of lateral roots in monocots. The significant induction of several ERFs and one ETHYLENE-INSENSITIVE 3 (EIN3) transcription factor (Bd1g63780v3) (a key TF promoting ethylene regulated gene expression) during T4 when more branched roots are formed (Fig. 1D), might indicate a positive role for this pathway in root branching in Brachypodium, as opposed to what has been suggested in Arabidopsis.

Our dataset revealed a significant upregulation of four WRKY TFs from T1 to T4. The bulk of WRKY TF experiments have focused on Arabidopsis and were shown to be involved in various stress responses^77,78^. In addition to stress, however, it was shown that the Arabidopsis TF *WRKY76* is involved in root development and phosphate acquisition^79^, while overexpression of *WRKY46* led to enhanced lateral root development^80^. Furthermore, the Arabidopsis *AtWRKY53* overexpression caused accelerated flowering^81^, suggesting a possible role of Brachypodium WRKY TFs in moving into reproductive stages at T4. Recent work in roots of *B. distachyon* seedlings demonstrated that multiple WRKY TF transcripts were present in the root and activated by application of the synthetic cytokinin 6-benzylaminopurine^82^. The trend of WRKY TFs upregulation from T1 to T4 indicated that this family of TFs could play a positive role in accelerating root growth, and further suggested their possible involvements in lateral root formation.

Similar to the WRKY family of TFs, we observed a family-wide upregulation of NAC TFs in T4 compared to T1. The NAC (NAM, ATAF1/2 and CUC2) proteins comprise a very large family of plant-specific TFs involved in multiple developmental processes^18^ including lateral root development^19,20^ and hormone signaling^19–22^. As with many TF families, NAC gene expression is altered by stress. However, transcriptome profiling of soybean^83^ and poplar^84^ revealed that NAC genes are predominantly expressed in root tissue and under non-stressed conditions, suggesting their possible role in global regulation of root development by interacting with internal growth factors. It was recently shown that TaRNAC1, a predominantly root-expressed NAC transcription factor from wheat, is not only involved in root length and biomass increase, but its overexpression can also lead to decreased time to shoot heading stage, and increase grain yield^85^. The Brachypodium NACs identified under this study may function similar to their wheat orthologs in the regulation of root growth, biomass production, and the timing of reproductive processes.

The GROWTH-REGULATING FACTOR1 (GRF) TF family was first identified in rice, and constitutes a small TF family of 12 genes in *B. distachyon*^86^. Our data identified 3 GRFs, all of which were significantly reduced at T4 compared to T1. The GRF TFs interact with GRF-interacting proteins (GIFs) and regulate gene expression in multiple tissues^87^. In rice, members of the GRF family are expressed mainly in actively growing organs but less induced in mature tissues^88^. The trend of high GRFs expression in young tissues that decreases during maturation is observed in other plant systems^86^. Overexpression of OsGRF1 in *Arabidopsis* resulted in delayed flowering times, indicating a role in the negative regulation of flowering^89^. Similarly, accelerated heading stage was correlated with a decrease in OsGRF1 expression in an *rhd1* mutant of rice^90^. Interestingly, however, RNAi mediated down-regulation of rice OsGRF1 displayed delayed growth and development, including delayed timing to the heading phase^90^. Our results propose a possible role of Brachypodium GRFs in root growth, and timing of the heading stage.

As described above, we showed a time-dependent induction of several TF families not only correlating with the root growth but also with the plant transition to reproductive growth stages (T4) in Brachypodium. As plant passes through the various developmental phases of its life cycle and continues to develop new organs, a number of morphological traits including size and shape of roots change in accordance with the developmental stage. As a result, different parts of a plant may exist in different growth stages and play role in phase transition^91^. Moreover, the developmental pathways leading to heading/flowering are regulated at numerous control points in different plant organs including roots in various plant species^92^, resulting in a diversity of timings to switch into reproductive phases. Both root and shoot organs need to meet the supply of the whole plant; this makes them dependent on each other functioning as an integrated system. This integration has been reported to be maintained throughout transition to the reproductive phase, by showing that the growth rate of both vegetative organs were equally affected by the withdrawal of resources to the emerging reproductive compartment^93^. This may explain the reason of the enrichment of several previously identified shoot-related vegetative-to-reproductive TFs in root system under this work. Following up on these observations with gene functional analysis, phenotypic screening of both above-and below-ground parts, and comparisons across Brachypodium genetic diversity will be an important future goal to develop a better understanding of TF-dependent root growth and developmental phase transition in grasses.

The growing number of genome sequences for numerous important plant species greatly facilitate promote the characterization of promoters and identification of novel motifs. The integrated genomics and to genome-wide transcriptomic analysis is a very efficient approach for prediction of *cis*-regulatory elements with various functionality^94^. This strategy have been employed in identifying several putative *cis*-elements within promoters from sucrose transporter genes and cold- and dehydration-responsive genes from Arabidopsis, rice, and soybean^95,96^. Similarly, large-scale prediction of numerous *cis*-acting elements involved in plant hormones^97^, calcium^98^, and biotic and abiotic stress responses were performed in Arabidopsis through global analysis of gene expression^99,100^. However, limited information available regarding root specific promoters^101,102^ and root-associated regulatory elements^103^ with the exception of a few well-known root specific motifs like ROOTMOTIFTAPOX1 element found in promoters of *rolD*^104^. In this study, the genome-wide analyses using available Brachypodium genome sequence and our time-dependent transcriptomic data revealed several putative *cis*-elements within promoters of the genes encoding TFs associated with root growth during plant developmental stages. Interestingly, one of the enriched motifs identified under cluster 1 (C1) of TFs (Fig. 5A,B), CYTCCTCYTCCC (all 18 promoter sequences in the cluster contributed to the construction of this motif), was associated with the Biological Function GO term root development (GO:0048364), and showed similarity to the ‘CTCTT’ signal sequence of OSE2ROOTNODULE as one of the few characterized root-specific motifs available in PLACE database^105^ (Table 1). Therefore, we suggest the 12 bp motif ‘CYTCCTCYTCCC’ as a potential candidate root regulatory element in Brachypodium with possible roles in activating and deactivating TFs likely involved in early developmental stages (Fig. 6), for further studies and validations. In order to prove the functional roles of the predicted *cis*-elements in this study, the presented short sequences need to be subjected to experimental verifications including loss-of function experiments by introducing point mutations into the target promoters (including the use of CRISPR technology^106^), and gain-of-function experiments by designing and constructing synthetic promoters^107,108^.

Roots are also major metabolic sinks for carbon acquired in terrestrial net primary productivity. The relative amount of biomass present in growing RSA is not fixed but may vary over time during plant development^109^. Carbon to nitrogen ratio in plant tissues during various developmental stages can actively adapt to the growth conditions and is a key parameter for estimating plant biomass allocation (Fig. 2B, Supplementary Table S1). Since we screened gene profiles of the whole-root system during time, the identified TFs and transcripts at later developmental stages (with increasing trend from T1 to T4) could be nominated as candidate Brachypodium root biomass-promoting genes. In addition to TFs, our whole root transcriptome analysis revealed genes that regulating below-ground carbohydrate metabolism with possible function in root biomass production. In particular, we observed that trehalose biosynthesis genes, TPSs, were differentially regulated during root growth. The trehalose biosynthetic pathway consists of two sequential enzymatic steps, wherein the metabolite intermediate trehalose-6-phosphate (T6P) is first formed by TPSs^49^, and then consumed by TPPs to generate trehalose^50^. The intermediate T6P (as a signal metabolite) is known to be an important signal in determining carbon utilization^110,111^, mainly by regulating sucrose consumption in developing sink organs including roots^112^. Our results suggest that differential regulation of trehalose biosynthesis genes and therefore possible altered T6P levels could play a role in root growth and biomass accumulation in Brachypodium. Although many biological processes including the whole-plant metabolism are affected by plant aging (without being directly involved in growth and development), the outcomes of this study provide a comprehensive platform for potential root developmental gene and promoter targets which can be employed by the genome editing and genetic engineering approaches for functional analyses and eventually for improving root growth parameters and biomass productivity in grasses in future works.

## Methods

### Plant growth conditions, root phenotyping and sampling

*Brachypodium distachyon* Bd21 was cultivated in growth chambers (Percival Scientific, Inc) in 9 cm size pots containing commercial soil (Sun Gro Horticulture, Metro-Mix 360 growth mix) under 16 h /8 h light-dark regime. Growth chamber settings included a light intensity of 250 µmol m^−2^ s^−1^, temperature of 24 °C day/18 °C night, and relative humidity of 60%. Plants were supplied with 50 ml water per pot^113^ every four days during the entire experimental period. Root phenotypic changes were assessed at 18 (T1), 25 (T2), 32 (T3), and 36 (T4) days post sowing (DPS). In each sampling-time point the entire root system was gently and quickly soaked and washed with water^7^, imaged, excised from the stem, flash frozen in liquid nitrogen, and stored at -80 °C for further analysis.

### Carbon and nitrogen measurement

The relative content of total carbon and total nitrogen (C/N ratio) was measured as described^114^ using freeze-dried, powdered samples by a VarioEL Cube Elemental Analyzer (Elementar Analysensysteme GmbH, Langenselbold, Germany). Statistical analysis was performed using a Kurskal-Wallis ANOVA, followed by pairwise Dunn’s tests^115^.

### RNA isolation and library construction

Liquid nitrogen, mortar and pestle were used to homogenize frozen root tissue. Approximately 100 mg of the resulting root homogenate from each sample was then subjected to RNA isolation using GeneJET Plant RNA Purification Kit (ThermoFisher Scientific, Cat# K0801) according to manufacturer’s protocols. All further processing was performed by the genomics core facility at Washington State University in Spokane, Washington according to Ingiosi *et al*. 2019 with minor modifications^116^. Total RNA integrity was assessed using Fragment Analyzer (Advanced Analytical Technologies, Ankeny, IA) with the High Sensitivity RNA Analysis Kit using manufacturer’s protocol. RNA samples without degradation were used for library preparation using the TruSeq Stranded RNA Library Prep Kit (Illumina, San Diego, CA). RNA Library size was assessed by Fragment Analyzer with the High Sensitivity NGS Fragment Analysis Kit, and library concentration was determined by StepOnePlus Real-Time PCR System (ThermoFisher Scientific, San Jose, CA) with the KAPA Library Quantification Kit (Kapabiosystems, Wilmington, MA) according to manufacturer’s protocols. Libraries were then diluted to 2 nM with RSB (10 mM Tris-HCl, pH8.5), denatured with 0.1 N NaOH, and eighteen pM was clustered in a flow cell using HiSeq Cluster Kit v4 on a cBot (Illumina), and loaded onto a HiSeq. 2500 (HiSeq SBS Kit v4, Illumina) according to manufacturer’s protocol. Paired-end sequencing was performed with a read length of 50 bp. Resulting BCL files were then converted to FASTQ files using bcl2fastq2.17.1.14. The adapters were trimmed from the FASTQ files during the conversion.

### Data quality control, alignment, and normalization

Trimmomatic (v0.38)^117^ was used to remove reads with a quality score less than 28 while maintaining a minimum read length of 34 bases. The effectiveness of this procedure was evaluated with FastQC^118^. Paired-end reads were then aligned using Hisat2 (v2.1.0)^119,120^ with an index created from the *Brachypodium distachyon* genome (v3.0) retrieved from EnsemblPlants^121^. The resulting alignment files were converted to the proper format with Samtools (v1.9)^122^ followed by transcript assembly and table count generation using StringTie (v1.3.5)^120^. Gene-level read counts were extracted using the Python script provided in the StringTie package (prepDE.py) and DESeq. 2^29^ normalized. We then pre-filtered and removed genes with very low read counts totaling <240 across all samples (DESeq. 2 rowSums function).

### qRT-PCR

qRT-PCR experiments were performed by the genomics core facility at Washington State University in Spokane, Washington. Total RNA was collected from the same plants used for RNA seq experiments in biological triplicate (n = 3) and subjected to reverse transcription according to the High Capacity cDNA Reverse Transcription Kit (ThermoFisher) (For T1, we pooled two biological samples to increase the mass of the starting material for RNA extraction.) Quantitative PCR was conducted in a 96-well plate format (Applied Biosystems) on StepOnePlus Real-Time PCR system using Power SYBR Green PCR Master Mix and twenty nanograms of cDNA for each sample. Ten microliter reactions were set-up and performed as outlined by the manufacturer (Applied Biosystems). Relative quantities (RQ) for transcripts were calculated using the 2^−ΔCT^ method, where ΔCt is the difference between the Ct of a target gene and the Ct of the endogenous control. In all cases, the endogenous control used was BRADI_3g14040v3^123^. The StepOne Software v2.3 (Applied Biosystems) was used to determine amplification cycle thresholds, and all samples were run in triplicate. Primers used in this study can be found in supplemental Table S9.

### Promoter analysis

The 1,000 bp genomic sequences upstream of each TF in clusters C1-C4 were analyzed by the Multiple Em for Motif Elicitation (MEME)^38^. Motif discovery was performed in classic mode with zero or one occurrence per sequence, searching for 5 motifs per cluster. Motif widths were restricted to between 8 and 12 bp long, and the search was performed only on the given strand. All other search parameters were left as default settings. Motifs discovered in this fashion were imported into the Gene Ontology for Motifs (GOMo) v 5.0.5^39^. Identified motifs were further scanned for the presence of putative *cis*- regulatory elements identical with or similar to the motifs registered in PLACE, a database of plant *cis* -acting regulatory DNA elements^23^.

### Statistical and enrichment analyses

For Partial Least Squares Discriminant Analysis (PLS-DA) the data were variance stabilized^29,124^ . Genes with a variable of importance in projection (VIP) score >1.0 in the first principal component (PC1) of the PLS-DA were chosen for further analysis^125,126^. Further, differentially expressed genes (DEGs) were identified between T1 and T4 samples using a t-test and considered significant if their Benjamini-Hochberg^127^ (B-H) corrected p-values were <0.0145 with a 10% false discovery rate. Enrichment analysis was carried out using the Uniprot^128^ gene accessions in conjunction with the DAVID tool^36^ (https://david.ncifcrf.gov/home.jsp). We compared our dataset to the Brachypodium genome using the DAVID online tool. The DAVID tool calculates fold enrichment by taking [# GO term X]/[# GO terms in user-provided list] and then dividing that by [# GO term X]/[# GO terms in Brachypodium genome]. Only genes with a high VIP score were included in the ontology enrichment. The B-H corrected p-value is the result of a modified Fisher Exact Test (which is then corrected for multiple hypothesis testing), to determine if the number of genes of a given ontology within our gene list (all genes with a PLSDA component 1 VIP > 1.0) are significantly over-represented given their natural abundance within the host Brachypodium genome. Hierarchical clustering was performed using Pearson correlation and average linkage, and the optimal number of clusters was determined using the gap statistic method^129^. Z-scores were calculated for each gene using the formula Z-score = (x-μ)/σ, where x, μ, and σ correspond to normalized gene count, population mean, and population standard deviation, respectively. In each case, the population refers to the abundance estimations for each individual gene across all timepoints (i.e. z-score by row).

## Supplementary information

Supplementary Information.

Supplementary Information2.

Supplementary Information3.

Supplementary Information4.

Supplementary Information5.

Supplementary Information6.

Supplementary Information7.

## Data Availability

Raw and processed RNA-seq data have been deposited in NCBIs Gene Expression Omnibus (http://www.ncbi.nlm.nih.gov/geo/) with accession number GSE131582.
