# Supplementary material for: Dynamics of Global Gene Expression and Regulatory Elements in Growing Brachypodium Root System
Source: Sci Rep. 2020 Apr 27;10:7071. doi: 10.1038/s41598-020-63224-z (PMC7184759; doi:10.1038/s41598-020-63224-z)
Supplement: Supplementary file 1 — Supplementary Information. [file 41598_2020_63224_MOESM1_ESM.pdf]

Supp Fig S3

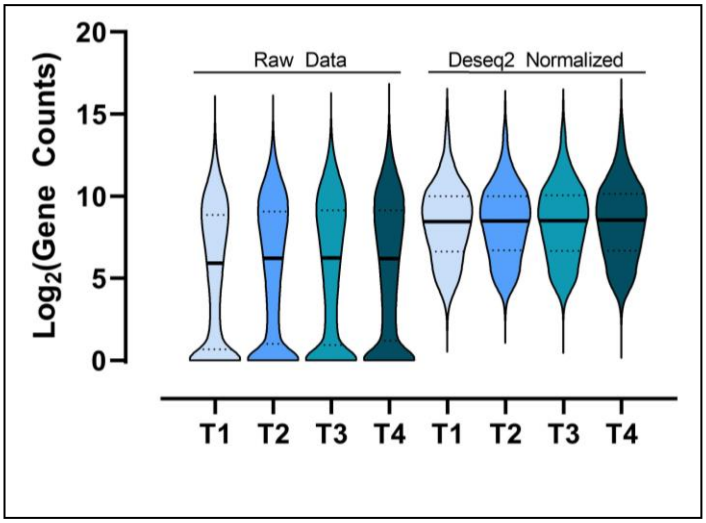

Supplemental Figure S3. Violin plots demonstrating the effectiveness of normalization and pre-filtering procedure. Prior to normalization the raw abundance estimates ("raw data", left) have large observable differences in total gene counts as well as the distribution of gene counts between samples. DESeq2 normalization resulted in a far more normal distribution in log2 space (right). Solid and dotted lines correspond to median and quartile values, respectively.

Supp Fig S5

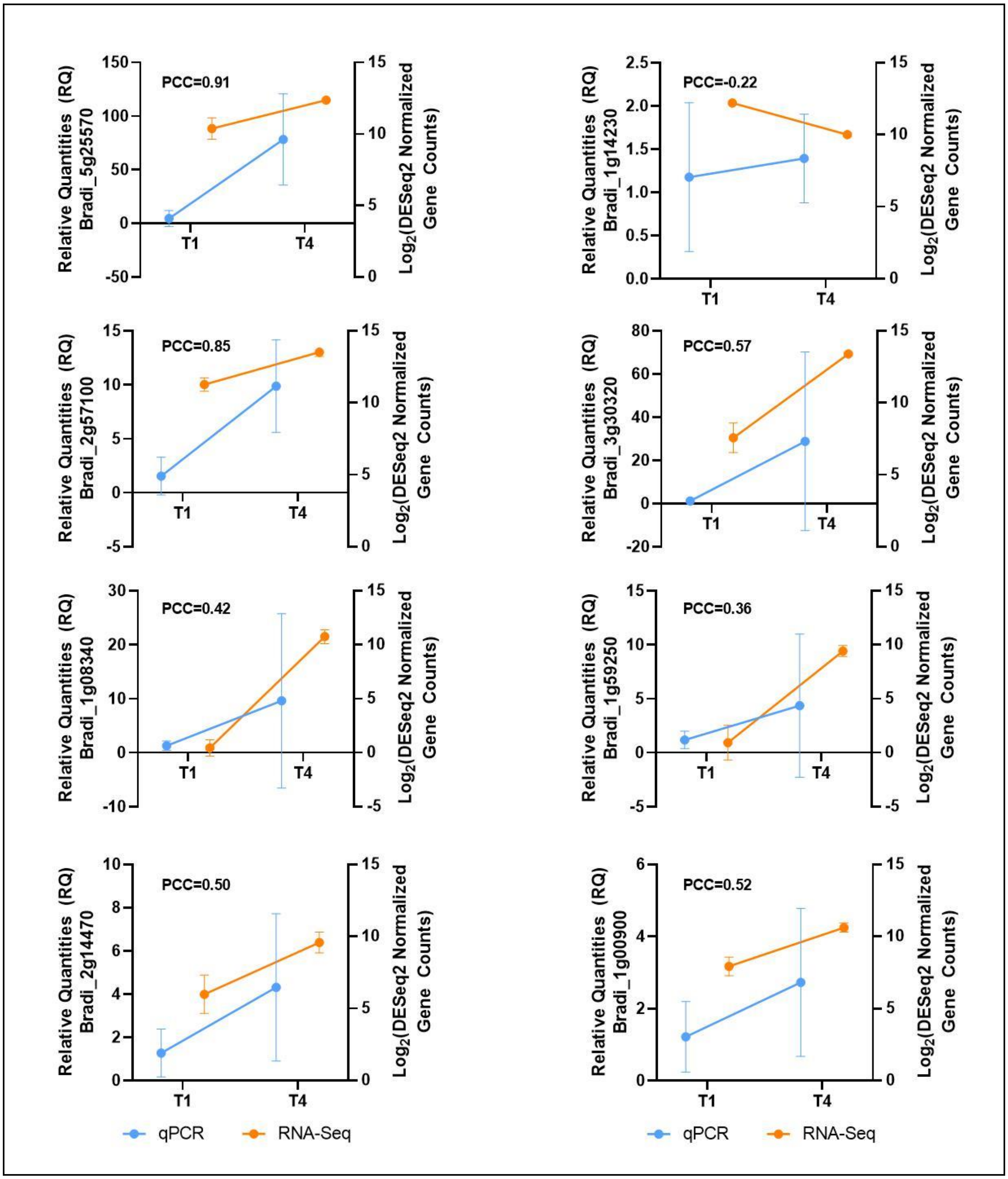

Supplemental Figure S5. RT-qPCR analysis of select genes in comparison with RNA-seq gene count estimations. RT-qPCR was performed in biological triplicate using Bradi_3g14040v3 as an endogenous control. RT-qPCR relative quantities (RQ) values for each gene are shown on the left y-axis in blue, and

log_2 scaled DESeq2 normalized gene counts are shown on the right y-axis in orange. Error bars represent the propagated standard deviation for qPCR, and standard deviation for log_2 normalized RNA-seq gene counts. PCC, Pearson Correlation Coefficient.

Supp Fig S8

Supplementary figure S8, this set of figures contains the output of the motif analysis performed by GOMo (part of the MEME-suite) using the motifs generated in Table 1 of the manuscript.

Cluster 1-1:

Investigated Motifs

Overview

Motif	Logo	Predictions	Top 5 specific predictions
1	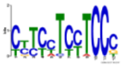	11	CC chloroplast<br>MF transcription factor activity<br>CC nucleus<br>CC plasma membrane<br>MF ATP binding

Motif 1

Top

GO terms are shown in grey if a more specific GO term was also significantly associated with this motif. The most specific GO terms are shown in black.
BP stands for biological process, CC stands for cellular component and MF stands for molecular function.

GO term	score	p-value	q-value	Specificity		GO name	Gene ID / Rank (25649 genes in total)
GO:0009507	9.668e-05	2.652e-07	1.659e-04	20%	CC	chloroplast	AT5G09760 (7), AT4G19920 (8), AT3G33530 (14), AT3G56140 (24), AT1G67810 (29), AT5G16890 (36), AT3G51510 (40), AT1G65230 (46), AT4G18480 (48), AT3G48110 (51), ...1957 more...
GO:0003700	2.441e-03	2.652e-07	1.659e-04	~83%	MF	transcription factor activity	AT5G60850 (6), AT1G06170 (27), AT4G29000 (32), AT5G19790 (57), AT5G58010 (72), AT1G32150 (90), AT1G58220 (117), AT2G36990 (164), AT1G77200 (173), AT5G05770 (174), ...1651 more...
GO:0005634	3.081e-03	2.652e-07	1.659e-04	~2%	CC	nucleus	AT5G64930 (23), AT1G27250 (52), AT5G19790 (57), AT5G58010 (72), AT3G58040 (116), AT5G44500 (159), AT3G11960 (161), AT1G26630 (163), AT1G77200 (173), AT3G60490 (188), ...1420 more...
GO:0003677	3.496e-03	2.652e-07	1.659e-04	~3%	MF	DNA binding	AT5G60850 (6), AT2G18090 (20), AT1G06170 (27), AT5G19790 (57), AT5G58010 (72), AT1G58220 (117), AT2G36990 (164), AT1G77200 (173), AT5G05770 (174), AT3G60490 (188), ...1217 more...
GO:0005886	8.383e-03	2.652e-07	1.659e-04	~1%	CC	plasma membrane	AT3G07040 (5), AT4G24100 (9), AT1G65040 (56), AT3G08600 (79), AT3G47950 (123), AT3G15180 (127), AT3G23750 (133), AT2G36910 (192), AT4G33625 (208), AT2G02400 (247), ...1175 more...
GO:0005524	8.834e-03	2.652e-07	1.659e-04	100%	MF	ATP binding	AT1G74330 (95), AT3G50230 (132), AT4G18640 (138), AT1G67510 (191), AT1G49270 (293), AT2G01950 (317), AT4G31180 (319), AT2G16440 (347), AT1G09620 (388), AT2G25140 (638), ...393 more...
GO:0045449	1.427e-02	3.978e-06	2.134e-03	~6%	BP	regulation of transcription	AT5G60850 (6), AT1G06170 (27), AT4G29000 (32), AT5G58010 (72), AT1G60300 (175), AT1G31050 (184), AT1G68360 (185), AT3G15030 (215), AT1G54060 (220), AT4G09180 (295), ...536 more...
GO:0048364	2.263e-02	1.803e-05	8.463e-03	~22%	BP	root development	AT2G46990 (292), AT3G62100 (299), AT5G01490 (332), AT5G60200 (489), AT1G01950 (575), AT4G00730 (1034), AT1G70940 (1382), AT5G59030 (1621), AT1G53330 (1698), AT3G02260 (1950), ...79 more...
GO:0016123	2.484e-02	2.678e-05	1.117e-02	100%	BP	xanthophyll biosynthetic process	AT4G25700 (1558), AT3G03710 (1714), AT5G67030 (1874), AT5G52570 (3667), AT5G57030 (10283), AT1G31800 (19708)
GO:0006468	3.585e-02	1.252e-04	4.700e-02	~18%	BP	protein amino acid phosphorylation	AT1G74330 (95), AT3G50230 (132), AT4G18640 (138), AT1G67510 (191), AT4G23650 (265), AT5G57610 (275), AT1G49270 (293), AT2G01950 (317), AT4G09570 (394), AT2G31500 (467), ...246 more...
GO:0005515	3.686e-02	1.395e-04	4.761e-02	~0%	MF	protein binding	AT4G34470 (1), AT5G41430 (2), AT5G41440 (3), AT5G45770 (4), AT3G07040 (5), AT1G72310 (45), AT1G65040 (56), AT3G06140 (87), AT4G19150 (111), AT3G58040 (116), ...1137 more...

Cluster 1-2:

Investigated Motifs

Overview

Motif	Logo	Predictions	Top 5 specific predictions
2	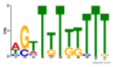	1	MF transcription factor activity

Motif 2

Top

GO terms are shown in grey if a more specific GO term was also significantly associated with this motif. The most specific GO terms are shown in black.
BP stands for biological process, CC stands for cellular component and MF stands for molecular function.

GO term	score	p-value	q-value	Specificity		GO name	Gene ID / Rank (25649 genes in total)
GO:0003700	1.294e-03	2.652e-07	9.992e-04	~83%	MF	transcription factor activity	AT1G60250 (1), AT4G00150 (3), AT1G01260 (7), AT3G17100 (17), AT4G01540 (19), AT1G06170 (21), AT1G21450 (23), AT1G22190 (38), AT4G39410 (47), AT2G23760 (55), ...1651 more...

Cluster 2-1:

Investigated Motifs

Overview

Motif	Logo	Predictions	Top 5 specific predictions
1	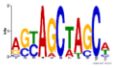	1	MF transcription factor activity

Motif 1

Top

GO terms are shown in grey if a more specific GO term was also significantly associated with this motif. The most specific GO terms are shown in black.
BP stands for biological process, CC stands for cellular component and MF stands for molecular function.

GO term	score	p-value	q-value	Specificity		GO name	Gene ID / Rank (25649 genes in total)
GO:0003700	5.627e-04	2.652e-07	1.000e-03	~83%	MF	transcription factor activity	AT1G66600 (12), AT4G38340 (63), AT1G47655 (69), AT1G13300 (78), AT1G17460 (131), AT3G50330 (134), AT4G34590 (146), AT1G76510 (167), AT5G45050 (230), AT5G58850 (250), ...1651 more...

Cluster 3-1:

Investigated Motifs

Overview

Motif	Logo	Predictions	Top 5 specific predictions
1	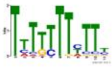	12	MF transcription factor activity<br>CC plasma membrane<br>CC nucleus<br>BP circadian rhythm<br>BP response to jasmonic acid stimulus

Motif 1

Top

GO terms are shown in grey if a more specific GO term was also significantly associated with this motif. The most specific GO terms are shown in black.
BP stands for biological process, CC stands for cellular component and MF stands for molecular function.

GO term	score	p-value	q-value	Specificity		GO name	Gene ID / Rank (25649 genes in total)
GO:0003700	9.870e-06	2.652e-07	1.661e-04	~83%	MF	transcription factor activity	AT3G06490 (4), AT1G18400 (15), AT3G55370 (38), AT3G53920 (77), AT2G25900 (110), AT4G39410 (126), AT4G36730 (130), AT5G49240 (132), AT5G09460 (133), AT4G30980 (135), ...1651 more...
GO:0045449	9.662e-04	2.652e-07	1.661e-04	~6%	BP	regulation of transcription	AT1G18400 (15), AT2G25900 (110), AT5G09460 (133), AT4G30980 (135), AT4G12620 (138), AT5G57150 (146), AT5G41030 (198), AT2G21320 (218), AT5G07690 (219), AT5G65100 (293), ...536 more...
GO:0005886	1.487e-03	2.652e-07	1.661e-04	~1%	CC	plasma membrane	AT3G59350 (11), ATPDIL1-3 (30), AT2G34480 (44), AT4G39350 (63), AT1G64650 (69), AT1G28290 (98), AT5G01890 (100), AT3G23600 (115), AT5G40370 (131), AT4G35240 (151), ...1175 more...
GO:0016301	3.636e-03	2.652e-07	1.661e-04	~1%	MF	kinase activity	AT3G05650 (7), AT2G20300 (9), AT3G59350 (11), AT1G16760 (37), AT1G51790 (67), AT5G01890 (100), AT1G64210 (127), AT2G23950 (129), AT5G45810 (140), AT5G51830 (155), ...899 more...
GO:0003677	3.797e-03	2.652e-07	1.661e-04	~3%	MF	DNA binding	AT3G06490 (4), AT3G55370 (38), AT3G53920 (77), AT2G38810 (91), AT5G24850 (95), AT2G25900 (110), AT3G18090 (118), AT4G30980 (135), AT4G12620 (138), AT5G41020 (141), ...1217 more...
GO:0005634	4.989e-03	2.652e-07	1.661e-04	~2%	CC	nucleus	AT3G55370 (38), AT4G31580 (64), AT2G31300 (82), AT5G37930 (83), AT3G23600 (115), AT1G80370 (121), AT4G12620 (138), AT5G56000 (159), AT3G23890 (175), AT1G68550 (179), ...1420 more...
GO:0007623	1.522e-02	3.182e-06	1.708e-03	~6%	BP	circadian rhythm	AT4G08920 (235), AT4G36240 (259), AT1G59940 (469), AT4G24470 (604), AT1G10470 (755), AT2G41310 (862), AT2G44680 (915), AT1G68830 (1231), AT3G46640 (1324), AT5G25830 (1458), ...35 more...
GO:0009753	1.858e-02	7.160e-06	3.363e-03	~45%	BP	response to jasmonic acid stimulus	AT3G06490 (4), AT1G28290 (98), AT4G34710 (117), AT5G07690 (219), AT5G07700 (283), AT4G22880 (373), AT3G16470 (418), AT1G06160 (421), AT4G23100 (465), AT2G31180 (531), ...110 more...
GO:0007169	2.072e-02	1.034e-05	4.318e-03	~12%	BP	transmembrane receptor protein tyrosine kinase signaling pathway	AT5G01890 (100), AT1G64210 (127), AT3G08680 (223), AT2G25790 (254), AT1G28440 (321), AT2G02780 (331), AT3G03770 (396), AT3G02880 (731), AT1G51830 (1234), AT3G20190 (1466), ...120 more...
GO:0007010	2.623e-02	3.182e-05	1.196e-02	~2%	BP	cytoskeleton organization	AT1G68060 (84), AT4G29350 (379), AT2G19760 (483), AT5G61980 (665), AT2G41740 (1723), AT2G29890 (1945), AT4G29340 (3726), AT1G14840 (4678), AT5G56600 (5799), AT3G53750 (6612), ...11 more...
GO:0006355	2.903e-02	4.747e-05	1.622e-02	~8%	BP	regulation of transcription, DNA-dependent	AT3G06490 (4), AT3G53920 (77), AT4G34710 (126), AT1G68550 (179), AT1G30330 (215), AT5G07690 (219), AT4G31650 (227), AT1G74650 (253), AT5G07700 (283), AT3G57600 (300), ...622 more...
GO:0004674	3.365e-02	8.433e-05	2.641e-02	~11%	MF	protein serine/threonine kinase activity	AT5G01890 (100), AT1G64210 (127), AT3G08680 (223), AT2G25790 (254), AT1G28440 (321), AT2G26980 (330), AT2G02780 (331), AT3G03770 (396), AT1G66750 (538), AT3G02880 (731), ...164 more...

Cluster 3-2:

Investigated Motifs

Overview

Motif	Logo	Predictions	Top 5 specific predictions
2	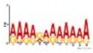	33	MF transcription factor activity<br>CC nucleus<br>CC plasma membrane<br>BP regulation of transcription, DNA-dependent<br>MF protein serine/threonine kinase activity

Motif 2

Top

GO terms are shown in grey if a more specific GO term was also significantly associated with this motif. The most specific GO terms are shown in black.
BP stands for biological process, CC stands for cellular component and MF stands for molecular function.

GO term	score	p-value	q-value	Specificity		GO name	Gene ID / Rank (25649 genes in total)
GO:0003700	2.892e-14	2.652e-07	6.555e-05	~83%	MF	transcription factor activity	AT4G01520 (8), AT3G47600 (21), AT3G55370 (28), AT5G40330 (38), AT4G36260 (39), AT2G45160 (40), AT1G69310 (41), AT5G43270 (53), AT1G28420 (57), AT4G14770 (59), ...1651 more...
GO:0005634	1.390e-08	2.652e-07	6.555e-05	~2%	CC	nucleus	AT3G55370 (28), AT2G46020 (30), AT3G13960 (36), AT5G40330 (38), AT4G36260 (39), AT1G13740 (46), AT4G17950 (50), AT5G43270 (53), AT1G28420 (57), AT3G07650 (67), ...1420 more...
GO:0003677	1.212e-07	2.652e-07	6.555e-05	~3%	MF	DNA binding	AT1G63480 (16), AT3G47600 (21), AT3G55370 (28), AT2G46020 (30), AT5G40330 (38), AT4G17950 (50), AT5G43270 (53), AT5G39660 (64), AT1G32240 (65), AT4G32730 (72), ...1217 more...
GO:0045449	6.103e-06	2.652e-07	6.555e-05	~6%	BP	regulation of transcription	AT3G47600 (21), AT5G40330 (38), AT2G45160 (40), AT5G43270 (53), AT2G35350 (56), AT4G14770 (59), AT5G39660 (64), AT1G32240 (65), AT3G07650 (67), AT4G32730 (72), ...536 more...
GO:0005886	9.998e-06	2.652e-07	6.555e-05	~1%	CC	plasma membrane	AT2G30740 (1), AT4G35240 (27), AT3G59350 (33), AT1G69270 (43), AT5G45470 (63), AT1G80660 (83), AT2G16250 (105), AT4G18820 (123), AT5G10450 (133), AT3G51390 (142), ...1175 more...
GO:0006355	4.700e-05	2.652e-07	6.555e-05	~8%	BP	regulation of transcription, DNA-dependent	AT1G69310 (41), AT4G17950 (50), AT1G28420 (57), AT4G32730 (72), AT5G60690 (81), AT1G50640 (91), AT5G60450 (97), AT5G67190 (109), AT4G34400 (118), AT3G06490 (168), ...622 more...
GO:0004674	2.355e-04	2.652e-07	6.555e-05	~11%	MF	protein serine/threonine kinase activity	AT5G58140 (31), AT1G48480 (95), AT2G16250 (105), AT3G20190 (145), AT5G10020 (187), AT5G01890 (314), AT4G20140 (318), AT3G17840 (489), AT5G58300 (564), AT3G08680 (586), ...164 more...
GO:0016301	3.003e-04	2.652e-07	6.555e-05	~1%	MF	kinase activity	AT2G30740 (1), AT5G58140 (31), AT3G59350 (33), AT1G69270 (43), AT1G48480 (95), AT4G33080 (102), AT3G20190 (145), AT2G07180 (154), AT3G62220 (156), AT1G16760 (159), ...899 more...
GO:0007169	3.328e-04	2.652e-07	6.555e-05	~12%	BP	transmembrane receptor protein tyrosine kinase signaling pathway	AT1G48480 (95), AT2G16250 (105), AT3G20190 (145), AT5G10020 (187), AT5G01890 (314), AT4G20140 (318), AT3G17840 (489), AT5G58300 (564), AT3G08680 (586), AT3G02880 (656), ...120 more...
GO:0006468	1.278e-03	2.652e-07	6.555e-05	~18%	BP	protein amino acid phosphorylation	AT1G48480 (95), AT2G16250 (105), AT3G20190 (145), AT5G10020 (187), AT5G12480 (279), AT5G01890 (314), AT4G20140 (318), AT5G23580 (331), AT4G18710 (475), AT3G17840 (489), ...246 more...
GO:0005515	1.341e-03	2.652e-07	6.555e-05	~0%	MF	protein binding	AT3G63530 (25), AT5G58550 (35), AT5G40330 (38), AT1G69270 (43), AT1G73950 (44), AT1G13740 (46), AT3G06660 (48), AT3G13380 (61), AT5G39660 (64), AT3G13460 (79), ...1137 more...
GO:0007623	4.016e-03	2.652e-07	6.555e-05	~6%	BP	circadian rhythm	AT4G18290 (231), AT3G22170 (734), AT1G59940 (852), AT5G17300 (969), AT4G08920 (1206), AT1G10470 (1318), AT4G24470 (1359), AT3G54810 (1802), AT3G24050 (1988), AT5G25830 (2104), ...35 more...
GO:0009908	9.162e-03	2.652e-07	6.555e-05	~13%	BP	flower development	AT4G37590 (73), AT5G37020 (209), AT5G45190 (238), AT2G38110 (243), AT1G30330 (302), AT2G21540 (345), AT1G24260 (629), AT3G54560 (928), AT1G67260 (1072), AT5G67440 (1111), ...70 more...
GO:0048366	9.792e-03	2.652e-07	6.555e-05	40%	BP	leaf development	AT3G13960 (36), AT5G45190 (238), AT4G19600 (329), AT3G15030 (466), AT5G20730 (842), AT2G28350 (918), AT5G27150 (962), AT2G31070 (1158), AT1G19220 (1267), AT3G61970 (1346), ...77 more...
GO:0005524	1.107e-02	2.652e-07	6.555e-05	100%	MF	ATP binding	AT1G27880 (10), AT2G46020 (30), AT1G48480 (95), AT2G16250 (105), AT3G22880 (132), AT3G20190 (145), AT5G10020 (187), AT2G01460 (217), AT2G07690 (223), AT5G44800 (258), ...393 more...
GO:0009414	1.432e-02	1.591e-06	3.687e-04	50%	BP	response to water deprivation	AT3G06760 (51), AT1G69700 (171), AT4G00430 (193), AT2G42620 (212), AT3G20310 (354), AT1G27320 (486), AT5G52310 (490), AT2G40220 (598), AT2G47800 (681), AT3G02480 (786), ...152 more...
GO:0005737	1.593e-02	3.447e-06	7.519e-04	~1%	CC	cytoplasm	AT2G15790 (19), AT3G13460 (79), AT3G04280 (104), AT5G10450 (133), AT5G53480 (147), AT2G32710 (192), AT5G67470 (216), AT2G07690 (223), AT2G41370 (274), AT3G04630 (287), ...460 more...
GO:0009736	1.690e-02	3.713e-06	7.647e-04	100%	BP	cytokinin mediated signaling pathway	AT3G04280 (104), AT1G27320 (486), AT1G49190 (558), AT5G10720 (574), AT1G19050 (837), AT1G59940 (852), AT4G31920 (1070), AT1G10470 (1318), AT2G25180 (1336), AT5G62920 (1353), ...29 more...
GO:0004722	1.839e-02	5.834e-06	1.138e-03	~54%	MF	protein serine/threonine phosphatase activity	AT3G47810 (45), AT2G35350 (56), AT3G07130 (162), AT1G72770 (233), AT5G10740 (268), AT3G06270 (407), AT3G17790 (477), AT2G42500 (481), AT1G67820 (512), AT3G12620 (521), ...133 more...
GO:0009944	2.066e-02	1.087e-05	2.016e-03	100%	BP	polarity specification of adaxial/abaxial axis	AT1G32240 (65), AT5G60690 (81), AT5G16560 (164), AT2G41370 (274), AT2G34710 (585), AT1G69180 (726), AT2G37630 (2454), AT1G23420 (3188), AT1G30490 (4477), AT1G65620 (4598), ...6 more...
GO:0009737	2.410e-02	2.121e-05	3.745e-03	60%	BP	response to abscisic acid stimulus	AT3G47600 (21), AT1G13740 (46), AT5G53160 (108), AT3G06490 (168), AT1G69700 (171), AT4G34710 (303), AT3G20310 (354), AT1G05470 (469), AT5G52310 (490), AT1G29395 (549), ...218 more...
GO:0005829	2.587e-02	2.997e-05	5.050e-03	~12%	CC	cytosol	AT2G46020 (30), AT5G10450 (133), AT5G10020 (187), AT3G01200 (227), AT1G69040 (235), AT4G35190 (260), AT4G31880 (338), AT2G42600 (394), AT5G58950 (409), AT3G26020 (417), ...419 more...
GO:0009751	2.870e-02	4.747e-05	7.415e-03	50%	BP	response to salicylic acid stimulus	AT3G47600 (21), AT1G74650 (563), AT2G36890 (1269), AT4G23270 (1281), AT1G14790 (1367), AT1G08810 (1374), AT5G49570 (1479), AT5G65790 (1608), AT5G46050 (1815), AT2G46410 (1817), ...103 more...
GO:0004672	2.876e-02	4.800e-05	7.415e-03	~5%	MF	protein kinase activity	AT3G13380 (61), AT5G54380 (201), AT3G01200 (227), AT4G24740 (388), AT4G18710 (475), AT5G01920 (613), AT5G55910 (636), AT3G48750 (685), AT1G09970 (769), AT2G26980 (808), ...139 more...
GO:0043565	2.944e-02	5.436e-05	8.062e-03	~12%	MF	sequence-specific DNA binding	AT3G19510 (334), AT4G40060 (400), AT1G69780 (999), AT3G54220 (1020), AT1G03970 (1865), AT2G46270 (2154), AT5G02030 (2457), AT4G01120 (2767), AT4G36730 (2915), AT5G61850 (2984), ...19 more...
GO:0009640	3.012e-02	5.887e-05	8.395e-03	~91%	BP	photomorphogenesis	AT3G55370 (28), AT5G23670 (307), AT1G20900 (713), AT3G48780 (1136), AT4G08920 (1206), AT1G69640 (1308), AT1G02090 (1626), AT2G46370 (1861), AT4G37580 (2276), AT2G45430 (2849), ...30 more...
GO:0009753	3.089e-02	6.470e-05	8.885e-03	~45%	BP	response to jasmonic acid stimulus	AT3G47600 (21), AT3G06490 (168), AT5G03280 (254), AT3G16470 (294), AT4G34710 (303), AT2G28900 (964), AT2G36890 (1269), AT1G08810 (1374), AT4G12030 (1389), AT5G46050 (1815), ...110 more...
GO:0007010	3.264e-02	7.955e-05	1.053e-02	~2%	BP	cytoskeleton organization	AT1G68060 (420), AT4G17220 (972), AT2G41740 (1134), AT4G29350 (1350), AT2G29890 (1912), AT2G19760 (2491), AT5G61980 (3018), AT4G29340 (3123), AT4G30160 (3317), AT1G24764 (4640), ...11 more...
GO:0009741	3.306e-02	8.433e-05	1.078e-02	~67%	BP	response to brassinosteroid stimulus	AT2G26710 (136), AT5G54380 (201), AT5G04240 (2481), AT3G61460 (2590), AT4G34160 (3587), AT4G00720 (4401), AT1G13260 (4466), AT2G44080 (4622), AT3G48430 (6158), AT3G50660 (6325), ...12 more...
GO:0010051	3.709e-02	1.313e-04	1.622e-02	~55%	BP	xylem and phloem pattern formation	AT4G36260 (39), AT5G60690 (81), AT4G08150 (96), AT5G16560 (164), AT3G51060 (849), AT1G15750 (1467), AT1G19850 (1516), AT1G04550 (1705), AT5G02030 (2457), AT4G39403 (3153), ...8 more...
GO:0016563	3.794e-02	1.458e-04	1.744e-02	~29%	MF	transcription activator activity	AT3G13960 (36), AT4G28500 (143), AT3G19510 (334), AT2G42380 (343), AT4G40060 (400), AT4G32040 (461), AT2G02540 (501), AT2G40220 (598), AT5G04410 (706), AT3G22170 (734), ...100 more...
GO:0006813	4.430e-02	2.829e-04	3.278e-02	~73%	BP	potassium ion transport	AT4G19960 (87), AT5G46240 (470), AT2G40540 (529), AT5G14880 (591), AT3G02850 (641), AT2G30070 (844), AT4G30940 (1062), AT3G02050 (1118), AT5G11800 (1473), AT5G41330 (2351), ...18 more...
GO:0010154	4.607e-02	3.363e-04	3.778e-02	~19%	BP	fruit development	AT1G14720 (68), AT5G60910 (595), AT2G28350 (918), AT4G34460 (1311), AT4G00180 (1879), AT5G02030 (2457), AT3G61880 (5523), AT4G36930 (5889), AT5G20490 (6173), AT1G17580 (6265), ...13 more...

Cluster 3-3:

Investigated Motifs

Overview

Motif	Logo	Predictions	Top 5 specific predictions
3	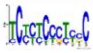	27	MF transcription factor activity<br>CC nucleus<br>MF protein binding<br>BP regulation of transcription, DNA-dependent<br>CC plasma membrane

Motif 3 Top

GO terms are shown in grey if a more specific GO term was also significantly associated with this motif. The most specific GO terms are shown in black.
BP stands for biological process, CC stands for cellular component and MF stands for molecular function.

GO term	score	p-value	q-value	Specificity		GO name	Gene ID / Rank (25649 genes in total)
GO:0003700	1.476e-09	2.652e-07	1.403e-04	~83%	MF	transcription factor activity	AT4G32040 (25), AT1G08010 (51), AT4G01520 (53), AT5G43290 (55), AT3G47600 (68), AT5G40330 (69), AT4G32730 (82), AT3G10040 (96), AT3G01600 (125), AT4G28530 (128), ...1651 more...
GO:0005634	7.308e-07	2.652e-07	1.403e-04	~2%	CC	nucleus	AT4G32040 (25), AT5G40330 (69), AT4G17950 (77), AT5G62920 (81), AT5G11530 (94), AT5G60880 (109), AT2G37520 (110), AT4G40060 (136), AT5G12400 (147), AT2G42500 (151), ...1420 more...
GO:0003677	1.715e-05	2.652e-07	1.403e-04	~3%	MF	DNA binding	AT3G47600 (68), AT5G40330 (69), AT4G17950 (77), AT1G63480 (80), AT4G32730 (82), AT2G37520 (110), AT5G12400 (147), AT1G28300 (183), AT5G66940 (185), AT3G54990 (186), ...1217 more...
GO:0045449	3.152e-04	2.652e-07	1.403e-04	~6%	BP	regulation of transcription	AT3G47600 (68), AT5G40330 (69), AT4G32730 (82), AT3G10040 (96), AT5G66940 (185), AT1G10610 (201), AT2G46510 (229), AT3G57920 (249), AT4G38000 (268), AT1G24625 (294), ...536 more...
GO:0005515	4.467e-04	2.652e-07	1.403e-04	~0%	MF	protein binding	AT5G13530 (11), AT5G22000 (12), AT4G18510 (23), AT2G33460 (27), AT1G55760 (39), AT5G40330 (69), AT5G58550 (104), AT5G25760 (118), AT4G26580 (126), AT4G13820 (170), ...1137 more...
GO:0006355	1.986e-03	2.652e-07	1.403e-04	~8%	BP	regulation of transcription, DNA-dependent	AT5G43290 (55), AT4G17950 (77), AT4G32730 (82), AT2G37520 (110), AT4G40060 (136), AT5G12400 (147), AT3G54990 (186), AT1G79840 (193), AT5G65310 (256), AT2G40620 (266), ...622 more...
GO:0005886	3.258e-03	2.652e-07	1.403e-04	~1%	CC	plasma membrane	AT2G30740 (18), AT1G64780 (33), AT1G76030 (67), AT2G04410 (74), AT3G51390 (91), AT2G23940 (97), AT3G03330 (124), AT2G22125 (132), AT2G43800 (139), AT1G71950 (205), ...1175 more...
GO:0009507	1.334e-02	3.182e-06	1.474e-03	20%	CC	chloroplast	AT2G21870 (16), AT1G01730 (41), AT5G19050 (61), AT2G39020 (63), AT1G76030 (67), AT1G50450 (100), AT1G50660 (115), AT4G26580 (126), AT5G42960 (133), AT5G09820 (141), ...1957 more...
GO:0003777	1.637e-02	5.038e-06	2.074e-03	80%	MF	microtubule motor activity	AT3G10180 (479), AT5G65930 (526), AT3G23670 (1109), AT1G21730 (1509), AT3G45850 (1586), AT5G47820 (1763), AT5G27550 (2042), AT5G42490 (2479), AT5G60930 (2640), AT1G23220 (2661), ...56 more...
GO:0016563	2.080e-02	1.379e-05	5.108e-03	~29%	MF	transcription activator activity	AT4G32040 (25), AT4G40060 (136), AT3G13960 (157), AT5G65310 (256), AT5G53660 (444), AT5G47670 (545), AT2G02540 (708), AT2G47460 (1259), AT3G48590 (1301), AT2G22840 (1355), ...100 more...
GO:0010158	2.200e-02	1.856e-05	6.252e-03	100%	BP	abaxial cell fate specification	AT5G60450 (374), AT4G00180 (782), AT1G08465 (1164), AT2G45190 (1914), AT5G16560 (1988), AT1G68480 (3566), AT2G33860 (4263)
GO:0005524	2.431e-02	2.599e-05	8.023e-03	100%	MF	ATP binding	AT1G27880 (1), AT1G09970 (150), AT5G44800 (188), AT1G14000 (303), AT3G20040 (305), AT1G65070 (315), AT1G48480 (324), AT2G46020 (334), AT1G05120 (358), AT5G45800 (390), ...393 more...
GO:0009744	2.677e-02	3.713e-05	1.058e-02	~67%	BP	response to sucrose stimulus	AT5G03730 (290), AT5G20270 (512), AT4G30850 (553), AT5G41670 (804), AT1G69780 (1064), AT4G34590 (1786), AT2G30470 (2624), AT2G23460 (2853), AT5G03840 (3112), AT1G61800 (3189), ...24 more...
GO:0004842	2.831e-02	5.012e-05	1.251e-02	~67%	MF	ubiquitin-protein ligase activity	AT5G13530 (11), AT3G54650 (54), AT3G24800 (86), AT5G25760 (118), AT1G45050 (217), AT4G33160 (416), AT2G47700 (610), AT3G63530 (617), AT2G42620 (813), AT4G38600 (844), ...183 more...
GO:0009944	2.838e-02	5.065e-05	1.251e-02	100%	BP	polarity specification of adaxial/abaxial axis	AT1G23420 (450), AT1G69180 (1344), AT2G45190 (1914), AT5G16560 (1988), AT5G60690 (2067), AT2G41370 (2524), AT1G30490 (2553), AT3G57130 (3244), AT1G32240 (3907), AT1G65620 (4669), ...6 more...
GO:0009738	2.915e-02	5.675e-05	1.314e-02	100%	BP	abscisic acid mediated signaling pathway	AT5G13530 (11), AT1G18890 (107), AT5G65310 (256), AT1G69270 (277), AT2G28350 (355), AT3G11410 (1186), AT2G26300 (1254), AT2G41070 (1296), AT5G53160 (1362), AT3G57530 (1424), ...36 more...
GO:0010599	3.030e-02	6.603e-05	1.439e-02	100%	BP	production of lsiRNA involved in RNA interference	AT1G69440 (2762), AT5G20320 (3716), AT1G01040 (4548)
GO:0048364	3.192e-02	8.353e-05	1.719e-02	~22%	BP	root development	AT3G16785 (281), AT1G05630 (316), AT3G22400 (890), AT3G28860 (1004), AT2G46990 (1113), AT1G13290 (1403), AT1G23080 (1452), AT1G04550 (1739), AT4G16780 (1775), AT2G39800 (2052), ...79 more...
GO:0048481	3.419e-02	1.098e-04	2.063e-02	~86%	BP	ovule development	AT1G23420 (450), AT5G62230 (1041), AT1G43850 (1523), AT5G42630 (1699), AT1G24260 (1857), AT5G16560 (1988), AT5G41410 (2405), AT3G02310 (2500), AT5G62000 (3616), AT1G32240 (3907), ...21 more...
GO:0009749	3.433e-02	1.114e-04	2.063e-02	~64%	BP	response to glucose stimulus	AT5G41670 (804), AT2G26300 (1254), AT5G64750 (1592), AT2G23460 (2853), AT2G19450 (3019), AT1G61800 (3189), AT1G74670 (3642), AT2G40220 (4241), AT1G20950 (4305), AT2G28900 (4626), ...9 more...
GO:0010050	3.686e-02	1.477e-04	2.606e-02	100%	BP	vegetative phase change	AT2G15790 (73), AT5G60450 (374), AT1G69440 (2762), AT5G20320 (3716), AT2G33860 (4263), AT3G01090 (5139), AT5G23570 (23954)
GO:0048366	3.847e-02	1.806e-04	3.011e-02	40%	BP	leaf development	AT1G65470 (117), AT3G13960 (157), AT2G31070 (298), AT2G28350 (355), AT5G53660 (444), AT4G14720 (566), AT3G61630 (758), AT1G01160 (1247), AT2G22840 (1355), AT3G48430 (1464), ...77 more...
GO:0005737	3.884e-02	1.870e-04	3.011e-02	~1%	CC	cytoplasm	AT2G15790 (73), AT2G42500 (151), AT2G32710 (195), AT5G13570 (216), AT5G17020 (219), AT5G18580 (222), AT4G05520 (301), AT1G19640 (341), AT4G34940 (380), AT2G41860 (469), ...460 more...
GO:0035196	4.009e-02	2.116e-04	3.266e-02	~95%	BP	production of miRNAs involved in gene silencing by miRNA	AT4G20910 (1031), AT1G69440 (2762), AT1G09700 (3323), AT5G20320 (3716), AT1G01040 (4548), AT3G20550 (12901), AT3G03300 (13249), AT3G43920 (14928), AT3G49500 (20413)
GO:0016564	4.283e-02	2.726e-04	4.040e-02	~33%	MF	transcription repressor activity	AT1G15750 (607), AT2G36080 (649), AT1G04550 (1739), AT4G11660 (1939), AT4G32551 (2252), AT2G30470 (2624), AT5G63320 (3204), AT4G14550 (3683), AT3G20310 (3792), AT1G50640 (4385), ...27 more...
GO:0004674	4.394e-02	3.002e-04	4.277e-02	~11%	MF	protein serine/threonine kinase activity	AT5G58140 (70), AT3G05840 (121), AT1G09970 (150), AT5G03730 (290), AT1G48480 (324), AT5G45800 (390), AT5G01890 (462), AT1G63430 (639), AT5G62310 (739), AT5G47760 (767), ...164 more...
GO:0009908	4.573e-02	3.543e-04	4.861e-02	~13%	BP	flower development	AT2G38110 (161), AT4G31820 (938), AT5G67440 (1063), AT5G57360 (1215), AT3G23130 (1623), AT1G30330 (1657), AT1G67260 (1782), AT3G61970 (1803), AT1G24260 (1857), AT1G22770 (2129), ...70 more...

Cluster 3-4:

Investigated Motifs

Overview

Motif	Logo	Predictions	Top 5 specific predictions
4	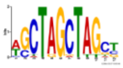	2	MF transcription factor activity<br>CC endomembrane system

Motif 4 Top

GO terms are shown in grey if a more specific GO term was also significantly associated with this motif. The most specific GO terms are shown in black.
BP stands for biological process, CC stands for cellular component and MF stands for molecular function.

GO term	score	p-value	q-value	Specificity		GO name	Gene ID / Rank (25649 genes in total)
GO:0003700	2.191e-03	2.652e-07	1.000e-03	~83%	MF	transcription factor activity	AT4G04890 (7), AT1G13300 (13), AT4G38340 (33), AT1G79840 (41), AT3G19070 (88), AT4G33450 (115), AT5G58850 (132), AT1G76510 (156), AT3G50330 (163), AT1G01720 (170), ...1651 more...
GO:0012505	2.197e-02	1.777e-05	3.350e-02	~2%	CC	endomembrane system	AT3G51644 (1), AT2G19893 (10), AT4G23690 (11), AT1G48440 (21), AT3G04170 (39), AT5G14105 (44), AT4G13968 (54), AT1G32970 (58), AT1G28695 (73), AT5G15725 (82), ...2957 more...

Cluster 3-5:

Investigated Motifs

Overview

Motif	Logo	Predictions	Top 5 specific predictions
5	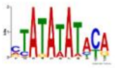	9	MF transcription factor activity<br>CC endomembrane system<br>BP response to auxin stimulus<br>MF polygalacturonase activity<br>BP oligopeptide transport

Motif 5

Top

GO terms are shown in grey if a more specific GO term was also significantly associated with this motif. The most specific GO terms are shown in black.
BP stands for biological process, CC stands for cellular component and MF stands for molecular function.

GO term	score	*p*-value	*q*-value	Specificity		GO name	Gene ID / Rank (25649 genes in total)
GO:0003700	1.982e-08	2.652e-07	1.663e-04	~83%	MF	transcription factor activity	AT3G30260 (2), AT1G13300 (3), AT2G36890 (57), AT1G74430 (70), AT1G43160 (76), AT1G73410 (101), AT5G67010 (111), AT2G46970 (118), AT1G71130 (139), AT4G30180 (158), ...1651 more...
GO:0012505	7.573e-06	2.652e-07	1.663e-04	~2%	CC	endomembrane system	AT3G48740 (7), AT3G29300 (11), AT2G33175 (17), AT1G06135 (18), AT2G03932 (36), AT1G23350 (43), AT3G47480 (48), AT4G16165 (50), AT3G47630 (58), AT3G28007 (75), ...2957 more...
GO:0045449	1.022e-03	2.652e-07	1.663e-04	~6%	BP	regulation of transcription	AT1G13300 (3), AT2G46970 (118), AT4G30180 (158), AT4G37850 (171), AT3G46090 (215), AT5G61620 (231), AT1G20910 (234), AT1G69690 (247), AT2G26580 (266), AT1G46480 (377), ...536 more...
GO:0009733	2.324e-03	2.652e-07	1.663e-04	~43%	BP	response to auxin stimulus	AT5G18060 (23), AT5G18050 (27), AT1G72430 (54), AT3G03847 (322), AT3G03850 (355), AT3G28860 (374), AT5G18020 (382), AT4G00880 (400), AT5G57560 (444), AT4G33880 (464), ...244 more...
GO:0003677	4.741e-03	2.652e-07	1.663e-04	~3%	MF	DNA binding	AT3G30260 (2), AT1G13300 (3), AT2G36890 (57), AT1G74430 (70), AT1G43160 (76), AT1G73410 (101), AT5G67010 (111), AT1G71130 (139), AT1G12630 (162), AT4G37850 (171), ...1217 more...
GO:0004650	8.602e-03	2.652e-07	1.663e-04	100%	MF	polygalacturonase activity	AT4G32380 (98), AT3G07840 (340), AT4G20050 (417), AT5G17200 (500), AT1G70500 (641), AT1G02790 (762), AT1G56710 (965), AT3G14040 (1173), AT4G23820 (1187), AT3G48950 (1496), ...61 more...
GO:0006857	1.913e-02	7.160e-06	3.850e-03	~45%	BP	oligopeptide transport	AT5G62680 (15), AT3G47960 (336), AT3G27020 (649), AT1G18880 (867), AT1G72130 (1150), AT1G72140 (1166), AT3G21670 (2169), AT3G45710 (2200), AT5G46050 (2415), AT5G01180 (2507), ...51 more...
GO:0005975	2.259e-02	1.538e-05	7.236e-03	~0%	BP	carbohydrate metabolic process	AT4G32380 (98), AT3G07840 (340), AT5G17200 (500), AT5G51820 (568), AT1G70500 (641), AT1G02790 (762), AT1G56710 (965), AT3G14040 (1173), AT4G23820 (1187), AT1G15380 (1204), ...106 more...
GO:0006355	3.032e-02	6.126e-05	2.562e-02	~8%	BP	regulation of transcription, DNA-dependent	AT2G36890 (57), AT1G74430 (70), AT1G43160 (76), AT1G73410 (101), AT5G67010 (111), AT1G71130 (139), AT1G12630 (162), AT5G62320 (177), AT2G40750 (183), AT1G35490 (192), ...622 more...

Cluster 4-1:

Investigated Motifs

Overview

Motif	Logo	Predictions	Top 5 specific predictions
1	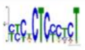	33	MF transcription factor activity<br>CC nucleus<br>CC plasma membrane<br>BP regulation of transcription, DNA-dependent<br>MF protein serine/threonine kinase activity

Motif 1

GO terms are shown in grey if a more specific GO term was also significantly associated with this motif. The most specific GO terms are shown in black.
BP stands for biological process, CC stands for cellular component and MF stands for molecular function.

GO term	score	p-value	q-value	Specificity	GO name	Gene ID / Rank (25649 genes in total)
GO:0003700	8.936e-13	2.652e-07	8.172e-05	~83%	MF transcription factor activity	AT4G01520 (9), AT3G47600 (11), AT5G40330 (14), AT4G32730 (20), AT3G10040 (25), AT4G40060 (39), AT1G27660 (44), AT1G77950 (58), AT2G25180 (66), AT4G28530 (70), ...1651 more...
GO:0005634	1.365e-07	2.652e-07	8.172e-05	~2%	CC nucleus	AT5G40330 (14), AT4G17950 (16), AT4G40060 (39), AT2G42500 (42), AT3G13960 (54), AT2G32710 (61), AT2G25180 (66), AT3G07590 (78), AT2G18960 (87), AT2G43370 (101), ...1420 more...
GO:0003677	1.083e-06	2.652e-07	8.172e-05	~3%	MF DNA binding	AT3G47600 (11), AT5G40330 (14), AT4G17950 (16), AT1G63480 (18), AT4G32730 (20), AT1G50620 (72), AT4G34400 (77), AT1G28300 (94), AT5G43270 (104), AT4G38000 (105), ...1217 more...
GO:0005886	5.339e-05	2.652e-07	8.172e-05	~1%	CC plasma membrane	AT2G30740 (1), AT3G51390 (22), AT3G03330 (29), AT2G43800 (31), AT3G07590 (78), AT1G76030 (83), AT4G05520 (84), AT2G18960 (87), AT5G55480 (89), ATVAMP711 (112), ...1175 more...
GO:0006355	1.515e-04	2.652e-07	8.172e-05	~8%	BP regulation of transcription, DNA-dependent	AT4G17950 (16), AT4G32730 (20), AT4G40060 (39), AT1G50620 (72), AT4G34400 (77), AT2G28350 (119), AT5G65050 (125), AT5G59780 (127), AT4G32800 (132), AT5G43290 (172), ...622 more...
GO:0045449	1.721e-04	2.652e-07	8.172e-05	~6%	BP regulation of transcription	AT3G47600 (11), AT5G40330 (14), AT4G32730 (20), AT3G10040 (25), AT1G24625 (92), AT5G43270 (104), AT4G38000 (105), AT5G65050 (125), AT2G45160 (135), AT1G68670 (145), ...536 more...
GO:0004674	1.294e-03	2.652e-07	8.172e-05	~11%	MF protein serine/threonine kinase activity	AT5G58140 (15), AT1G09970 (45), AT5G03730 (90), AT5G01890 (139), AT1G48480 (146), AT5G01950 (255), AT5G45800 (281), AT4G37250 (293), AT2G23300 (388), AT5G47760 (404), ...164 more...
GO:0048366	1.942e-03	2.652e-07	8.172e-05	40%	BP leaf development	AT3G13960 (54), AT2G28350 (119), AT2G31070 (161), AT3G61630 (178), AT4G14720 (402), AT5G53660 (435), AT1G07630 (523), AT1G01160 (573), AT3G61970 (1257), AT2G22840 (1289), ...77 more...
GO:0005515	2.076e-03	2.652e-07	8.172e-05	~0%	MF protein binding	AT5G40330 (14), AT5G58550 (27), AT1G55760 (50), AT2G32400 (69), AT2G18960 (87), AT5G03730 (90), AT1G55310 (97), AT5G25760 (98), AT3G58720 (156), AT4G29240 (166), ...1137 more...
GO:0007623	6.745e-03	2.652e-07	8.172e-05	~6%	BP circadian rhythm	AT4G18290 (663), AT5G57360 (714), AT3G24050 (1349), AT4G08920 (1488), AT5G61380 (1604), AT3G54810 (1696), AT3G04910 (1777), AT1G22770 (1803), AT5G02840 (2166), AT2G46830 (2498), ...35 more...
GO:0003777	6.924e-03	2.652e-07	8.172e-05	80%	MF microtubule motor activity	AT1G21730 (638), AT5G65930 (847), AT3G45850 (894), AT2G36200 (1056), AT5G47820 (1272), AT3G23670 (1321), AT3G19050 (1634), AT5G60930 (1717), AT3G49650 (1843), AT3G16630 (1966), ...56 more...
GO:0006468	7.084e-03	2.652e-07	8.172e-05	~18%	BP protein amino acid phosphorylation	AT1G09970 (45), AT5G23580 (80), AT5G01890 (139), AT1G48480 (146), AT4G17640 (207), AT5G01950 (255), AT5G45800 (281), AT3G51850 (289), AT4G37250 (293), AT5G55910 (351), ...246 more...
GO:0016563	7.573e-03	5.304e-07	1.401e-04	~29%	MF transcription activator activity	AT4G40060 (39), AT3G13960 (54), AT2G02540 (221), AT5G65310 (364), AT5G53660 (435), AT1G63910 (551), AT2G47460 (903), AT4G01540 (986), AT5G42910 (1033), AT1G79180 (1080), ...100 more...
GO:0007169	7.915e-03	5.304e-07	1.401e-04	~12%	BP transmembrane receptor protein tyrosine kinase signaling pathway	AT1G09970 (45), AT5G01890 (139), AT1G48480 (146), AT5G01950 (255), AT5G45800 (281), AT4G37250 (293), AT2G23300 (388), AT5G16590 (579), AT1G63430 (651), AT4G34220 (694), ...120 more...
GO:0005524	1.137e-02	1.326e-06	3.269e-04	100%	MF ATP binding	AT1G27880 (4), AT1G09970 (45), AT5G44800 (114), AT2G46020 (118), AT5G01890 (139), AT1G48480 (146), AT3G22880 (152), AT5G01950 (255), AT5G45800 (281), AT5G63950 (282), ...393 more...
GO:0009944	1.186e-02	1.856e-06	4.290e-04	100%	BP polarity specification of adaxial/abaxial axis	AT1G69180 (395), AT1G30490 (609), AT1G32240 (1000), AT2G41270 (1489), AT3G57130 (2186), AT5G16560 (2319), AT5G60690 (2567), AT1G65620 (2991), AT2G45190 (4327), AT2G37630 (4881), ...6 more...
GO:0010051	1.843e-02	9.281e-06	2.019e-03	~55%	BP xylem and phloem pattern formation	AT1G15750 (219), AT1G20330 (603), AT1G04550 (626), AT4G08150 (764), AT3G51060 (902), AT4G36260 (1004), AT5G02030 (1183), AT5G16560 (2319), AT5G60690 (2567), AT1G19850 (3514), ...8 more...
GO:0045941	2.087e-02	1.326e-05	2.550e-03	~32%	BP positive regulation of transcription	AT4G40060 (39), AT3G55370 (121), AT2G02540 (221), AT5G65310 (364), AT3G50410 (888), AT3G51060 (902), AT5G04410 (1472), AT2G22430 (1616), AT3G19510 (1658), AT3G26744 (1800), ...22 more...
GO:0009744	2.108e-02	1.379e-05	2.550e-03	~67%	BP response to sucrose stimulus	AT5G03730 (90), AT1G69780 (357), AT2G30470 (1010), AT5G20270 (1123), AT5G41670 (1528), AT5G03840 (1775), AT1G61800 (2203), AT4G34590 (2361), AT1G74670 (2770), AT4G30850 (3266), ...24 more...
GO:0009733	2.110e-02	1.379e-05	2.550e-03	~43%	BP response to auxin stimulus	AT3G47600 (11), AT3G12955 (159), AT5G54500 (171), AT4G32690 (189), AT1G15750 (219), AT1G70000 (331), AT2G16580 (367), AT5G20820 (431), AT2G46990 (553), AT2G47000 (595), ...244 more...
GO:0016301	2.387e-02	2.360e-05	4.156e-03	~1%	MF kinase activity	AT2G30740 (1), AT5G58140 (15), AT3G62220 (24), AT4G33080 (40), AT1G09970 (45), AT5G23580 (80), AT5G03730 (90), AT5G45430 (131), AT5G01890 (139), AT1G48480 (146), ...899 more...
GO:0048481	2.443e-02	2.519e-05	4.235e-03	~86%	BP ovule development	AT5G41410 (476), AT1G43850 (710), AT5G42630 (974), AT1G32240 (1000), AT1G24260 (1311), AT5G07180 (2304), AT5G16560 (2319), AT1G25350 (2368), AT5G23260 (2857), AT2G25840 (3358), ...21 more...
GO:0010154	2.819e-02	4.879e-05	7.845e-03	~19%	BP fruit development	AT2G28350 (119), AT1G14720 (204), AT4G00180 (334), AT5G02030 (1183), AT4G02460 (1262), AT3G61880 (1697), AT5G60910 (1997), AT3G22200 (2163), AT4G34460 (2607), AT5G20490 (3872), ...13 more...
GO:0009908	3.225e-02	8.327e-05	1.283e-02	~13%	BP flower development	AT2G38110 (47), AT5G67440 (355), AT4G31820 (534), AT1G67260 (635), AT5G57360 (714), AT1G20330 (751), AT5G10510 (755), AT4G36920 (801), AT3G54720 (920), AT3G23130 (928), ...70 more...
GO:0009965	3.339e-02	9.573e-05	1.416e-02	~52%	BP leaf morphogenesis	AT2G31070 (161), AT1G69780 (357), AT4G01540 (986), AT3G13300 (1721), AT1G48410 (1738), AT4G18710 (1875), AT5G08370 (2276), AT2G19520 (2354), AT2G44950 (2482), AT4G36870 (2534), ...39 more...
GO:0010158	3.448e-02	1.103e-04	1.569e-02	100%	BP abaxial cell fate specification	AT4G00180 (334), AT5G60450 (601), AT1G08465 (941), AT5G16560 (2319), AT2G45190 (4327), AT2G33860 (5603), AT1G68480 (11686)
GO:0005737	3.511e-02	1.188e-04	1.627e-02	~1%	CC cytoplasm	AT2G15790 (13), AT2G42500 (42), AT2G32710 (61), AT4G05520 (84), AT5G47120 (194), AT5G17020 (205), AT5G53480 (242), AT5G13570 (272), AT1G35730 (297), AT4G23860 (311), ...460 more...
GO:0004672	3.550e-02	1.236e-04	1.632e-02	~5%	MF protein kinase activity	AT1G09970 (45), AT5G55910 (351), AT4G38470 (399), AT3G23750 (614), AT2G17700 (634), AT5G14640 (742), AT3G53030 (819), AT4G30520 (823), AT1G14000 (875), AT1G65800 (956), ...139 more...
GO:0043565	4.051e-02	2.198e-04	2.803e-02	~12%	MF sequence-specific DNA binding	AT4G40060 (39), AT1G69780 (357), AT5G65310 (364), AT1G03970 (753), AT5G02030 (1183), AT4G16780 (1480), AT2G22430 (1616), AT3G19510 (1658), AT5G61850 (1723), AT4G24540 (1960), ...19 more...
GO:0009736	4.305e-02	2.787e-04	3.347e-02	100%	BP cytokinin mediated signaling pathway	AT2G25180 (66), AT1G49190 (360), AT1G74890 (368), AT1G27320 (419), AT5G10720 (448), AT4G01540 (986), AT1G70510 (2057), AT1G19050 (2143), AT3G48100 (2921), AT5G62920 (2951), ...29 more...
GO:0016481	4.314e-02	2.806e-04	3.347e-02	~30%	BP negative regulation of transcription	AT5G08550 (124), AT4G38130 (911), AT3G23130 (928), AT4G32551 (1072), AT5G02030 (1183), AT4G16780 (1480), AT4G05420 (1928), AT5G18230 (1958), AT1G59750 (2169), AT2G37630 (4881), ...21 more...
GO:0000151	4.507e-02	3.445e-04	3.981e-02	~22%	CC ubiquitin ligase complex	AT3G24800 (21), AT3G62980 (628), AT1G55860 (1174), AT3G42830 (1681), AT4G02570 (1712), AT2G42620 (2176), AT1G70320 (3101), AT5G46210 (5401), AT3G53090 (7539), AT5G05560 (8733), ...7 more...
GO:0009640	4.646e-02	3.901e-04	4.371e-02	~91%	BP photomorphogenesis	AT3G55370 (121), AT3G28860 (696), AT3G54720 (920), AT4G14110 (926), AT2G37678 (967), AT2G46370 (1095), AT1G06040 (1141), AT4G08920 (1488), AT5G11260 (1656), AT1G14290 (2924), ...30 more...

Cluster 4-2:

Investigated Motifs

Overview

Motif	Logo	Predictions	Top 5 specific predictions
2	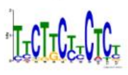	9	CC chloroplast<br>CC nucleus<br>CC plasma membrane<br>CC respiratory chain complex I<br>MF transcription factor activity

Motif 2

Top

GO terms are shown in grey if a more specific GO term was also significantly associated with this motif. The most specific GO terms are shown in black.
BP stands for biological process, CC stands for cellular component and MF stands for molecular function.

GO term	score	p-value	q-value	Specificity	GO name	Gene ID / Rank (25649 genes in total)
GO:0009507	8.026e-05	2.652e-07	3.312e-04	20%	CC chloroplast	AT1G11520 (12), AT5G40610 (18), AT5G07020 (41), AT2G30020 (92), AT1G22700 (95), AT1G32900 (105), AT4G33130 (116), AT5G55610 (124), AT5G10920 (131), AT1G35680 (151), ...1957 more...
GO:0005634	5.480e-03	2.652e-07	3.312e-04	~2%	CC nucleus	AT2G38940 (11), AT3G23890 (15), AT3G55320 (24), AT5G64960 (65), AT3G22170 (87), AT1G26960 (88), AT1G14130 (93), AT4G24020 (130), AT2G26990 (142), AT3G23050 (145), ...1420 more...
GO:0005886	6.040e-03	2.652e-07	3.312e-04	~1%	CC plasma membrane	AT4G23460 (5), AT1G77740 (63), AT5G10020 (83), AT1G34300 (84), AT4G10170 (107), AT1G63110 (133), AT2G31820 (154), AT1G27320 (172), AT3G06035 (175), AT4G29900 (197), ...1175 more...
GO:0045271	1.456e-02	4.243e-06	3.974e-03	~73%	CC respiratory chain complex I	AT2G02050 (696), AT5G67590 (759), AT5G40770 (1050), AT2G31490 (1069), AT2G02510 (1265), AT2G47690 (1397), AT1G67785 (1640), AT1G47260 (1715), AT1G76200 (2077), AT5G08530 (2341), ...35 more...
GO:0003700	2.026e-02	1.034e-05	6.458e-03	~83%	MF transcription factor activity	AT5G13180 (20), AT2G02450 (46), AT4G29000 (49), AT1G12860 (59), AT1G29950 (74), AT4G01460 (76), AT3G22170 (87), AT1G26960 (88), AT5G04840 (89), AT5G39700 (103), ...1651 more...
GO:0016301	2.048e-02	1.034e-05	6.458e-03	~1%	MF kinase activity	AT5G56040 (43), AT5G64960 (65), AT1G34300 (84), AT1G09600 (102), AT1G51790 (132), AT3G45390 (137), AT5G12480 (146), AT1G48480 (186), AT5G58300 (206), AT2G38910 (219), ...899 more...
GO:0004674	2.499e-02	2.493e-05	1.334e-02	~11%	MF protein serine/threonine kinase activity	AT5G56040 (43), AT5G10020 (83), AT3G49670 (122), AT5G45840 (134), AT1G48480 (186), AT5G47760 (202), AT5G58300 (206), AT2G16250 (235), AT1G53730 (317), AT1G21210 (497), ...164 more...
GO:0007169	2.927e-02	5.198e-05	2.434e-02	~12%	BP transmembrane receptor protein tyrosine kinase signaling pathway	AT5G56040 (43), AT5G10020 (83), AT3G49670 (122), AT5G45840 (134), AT1G48480 (186), AT5G58300 (206), AT2G16250 (235), AT1G53730 (317), AT5G14210 (510), AT2G23300 (513), ...120 more...
GO:0003723	3.612e-02	1.199e-04	4.990e-02	~2%	MF RNA binding	AT1G69250 (111), AT5G18810 (152), AT3G03920 (161), AT1G22240 (182), AT2G22100 (196), AT4G36690 (226), AT2G17510 (333), AT1G12800 (401), AT2G43370 (518), AT5G23080 (523), ...309 more...
